# Pathology-guided design of injectable hydrogels for precision therapy and cartilage regeneration in osteoarthritis

**DOI:** 10.1093/rb/rbag009

**Published:** 2026-01-25

**Authors:** Zhi Zheng, Jiahao Xie, Junfa Zeng, Zhan Kang, Zhenqiu Liu, Mengzhen Qi, Cuiyun Yu, Hua Wei

**Affiliations:** Hunan Province Cooperative Innovation Center for Molecular Target New Drug Study & School of Pharmaceutical Science, Hengyang Medical School, University of South China, 28 W Changsheng Road, Hengyang 421001, China; Hunan Province Cooperative Innovation Center for Molecular Target New Drug Study & School of Pharmaceutical Science, Hengyang Medical School, University of South China, 28 W Changsheng Road, Hengyang 421001, China; Hunan Province Cooperative Innovation Center for Molecular Target New Drug Study & School of Pharmaceutical Science, Hengyang Medical School, University of South China, 28 W Changsheng Road, Hengyang 421001, China; Hunan Province Cooperative Innovation Center for Molecular Target New Drug Study & School of Pharmaceutical Science, Hengyang Medical School, University of South China, 28 W Changsheng Road, Hengyang 421001, China; Hunan Province Cooperative Innovation Center for Molecular Target New Drug Study & School of Pharmaceutical Science, Hengyang Medical School, University of South China, 28 W Changsheng Road, Hengyang 421001, China; Hunan Province Cooperative Innovation Center for Molecular Target New Drug Study & School of Pharmaceutical Science, Hengyang Medical School, University of South China, 28 W Changsheng Road, Hengyang 421001, China; Hunan Province Cooperative Innovation Center for Molecular Target New Drug Study & School of Pharmaceutical Science, Hengyang Medical School, University of South China, 28 W Changsheng Road, Hengyang 421001, China; Affiliated Hospital of Hunan Academy of Chinese Medicine, Hunan Academy of Chinese Medicine, Changsha 410013, China; Hunan Province Cooperative Innovation Center for Molecular Target New Drug Study & School of Pharmaceutical Science, Hengyang Medical School, University of South China, 28 W Changsheng Road, Hengyang 421001, China

**Keywords:** cartilage damage, injectable hydrogel, precision medicine, pathological mechanisms, cartilage regeneration

## Abstract

Osteoarthritis (OA) is a highly prevalent degenerative joint disease whose complex pathological microenvironment and limited cartilage self-repair capacity have resulted in the absence of therapeutic approaches capable of simultaneously achieving structural reconstruction and functional recovery. Current clinical strategies face significant limitations, as conventional pharmacological treatments can only alleviate symptoms with accompanying systemic side effects, while surgical interventions often encounter challenges such as inadequate mechanical properties of repaired tissues and long-term degeneration. The precise functionalization of injectable hydrogels represents a key strategy for cartilage regeneration and the core challenge lies in integrating multiple material properties to design on-demand delivery platforms that can dynamically respond to complex pathological microenvironments *in vivo*. This review systematically elaborates on precision customization strategies for injectable hydrogels based on OA pathological mechanisms, focusing on how hydrogel design responds to pathological signals in the joint microenvironment to achieve on-demand and precise regulation of therapeutic agents including drugs, cells and genes. Beginning with cartilage structure and injury mechanisms, this article analyzes the limitations of existing pharmacological and surgical repair methods, then, elaborate on the multifunctional platform role of hydrogels in cartilage tissue engineering, including recent advances in mechanical design, drug loading/release behavior, inflammation regulation, stem cell delivery and gene-activated repair. Finally, it outlines challenges and future directions for smart hydrogels in cartilage regenerative medicine, aiming to provide a theoretical framework and technical pathway for integrating materials science with clinical medicine.

## Graphical abstract

**Figure:**
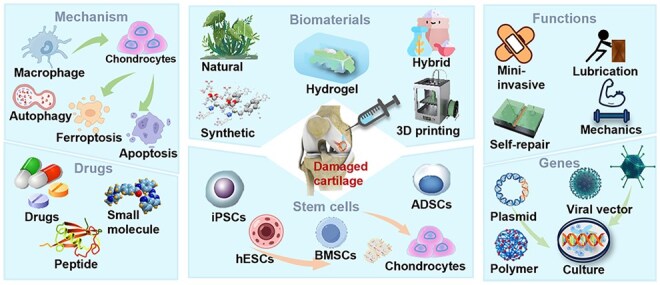

## Introduction

Cartilage possesses a limited capacity for spontaneous regeneration following injury, attributable to its avascular nature, low cellular density and diminished metabolic activity [1]. Abnormal or nonphysiological mechanical loading resulting from joint dysfunction can induce damage to the extracellular matrix (ECM), ultimately provoking an inflammatory response. Without timely intervention, acute cartilage injuries frequently progress to a state of persistent degradation, culminating in a chronic inflammatory condition [2]. Large focal cartilage defects further accelerate tissue-wide degeneration, increasing susceptibility to osteoarthritis (OA) and rheumatoid arthritis (RA). Globally, nearly 600 million individuals are affected by OA and RA collectively [3]. Although the precise pathological mechanisms of OA remain incompletely elucidated, mechanical damage is widely recognized as a major contributing factor. These conditions not only significantly impair patients’ quality of life but also pose a substantial socioeconomic burden, with annual costs exceeding USD 100 billion due to healthcare expenses and productivity losses [4, 5].

The therapeutic landscape for OA remains predominantly pharmacological, with conventional regimens focusing on symptom management through analgesic effects, inflammatory modulation and ECM degradation inhibition [5, 6]. Systemic pharmacotherapy often elicits dose-limiting adverse events, including gastrointestinal mucosal damage, ulcerogenesis, coagulopathies and cardiovascular sequelae [7]. For advanced cases, surgical interventions such as microfracture, osteochondral grafting and autologous chondrocyte implantation are implemented, yet these procedures frequently result in suboptimal cartilage integration and biomechanical instability [7, 8].

The difficulty in achieving effective cartilage repair arises from the complex interplay among structural defects, dysfunctional molecular signaling and biomechanical compromises [9, 10]. Structural abnormalities often impede successful surgical restoration, while dysregulated inflammatory and catabolic pathways undermine the efficacy of pharmacological treatments [10]. Moreover, functional failure at the osteochondral interface, particularly the imperfect recapitulation of the calcified cartilage layer, further complicates regenerative outcomes. It is this persistent inability to restore native tissue architecture and function that has motivated intensified research into the mechanisms behind failed cartilage regeneration. A deeper understanding of these processes is essential to transcend the limitations of current strategies and develop integrated, pathophysiology-informed therapeutic solutions.

Tissue engineering and biomaterial-based approaches have emerged as promising alternatives for cartilage regeneration by mimicking the native hierarchical architecture and biomechanical properties of articular cartilage [11–13]. A key advancement lies in the targeted delivery of bioactive molecules via engineered constructs, which enables more precise modulation of pathological mechanisms and improved functional outcomes. These capabilities have positioned bioengineering strategies as a viable pathway for addressing cartilage defects. Among these, injectable biomimetic hydrogels have garnered significant attention due to their capacity to replicate critical features of cartilage ECM [14, 15]. Their minimally invasive delivery, ability to conform to irregular defect geometries and *in situ* cross-linking to form stable three-dimensional (3D) networks not only facilitate surgical application but also enhance defect filling efficacy [16]. Furthermore, the tunable mechanical properties of these hydrogels allow matching the compressive modulus and lubricating functions of native cartilage, thereby mitigating joint stress and reducing wear-induced pain. A distinctive advantage is their capacity to immobilize therapeutic agents or cells through physical entrapment or covalent binding, enabling sustained, localized release that enhances regenerative efficacy. Complemented by progress in 3D printing technology over the past two decades, which has further expanded customization capabilities for cartilage scaffolds [17], these developments reflect substantial growth in the field.

Despite these developments, current research remains largely focused on isolated solutions and lacks an integrated multifaceted perspective that combines hydrogel design with the pathomechanisms of cartilage injury. This limitation underscores the need for a paradigm shift toward microenvironment-responsive intelligent biomaterials—a broader materials science domain that synergizes material innovation with pathological biology. By dynamically sensing and responding to disease-specific microenvironments (e.g. inflammatory cytokines, oxidative stress or mechanical overload), these smart materials transcend passive scaffolding to actively participate in regulating pathological processes [18]. OA serves as a prototypical example to illustrate the generalizability of this material-biology coupling design principle.

**Figure 1:**
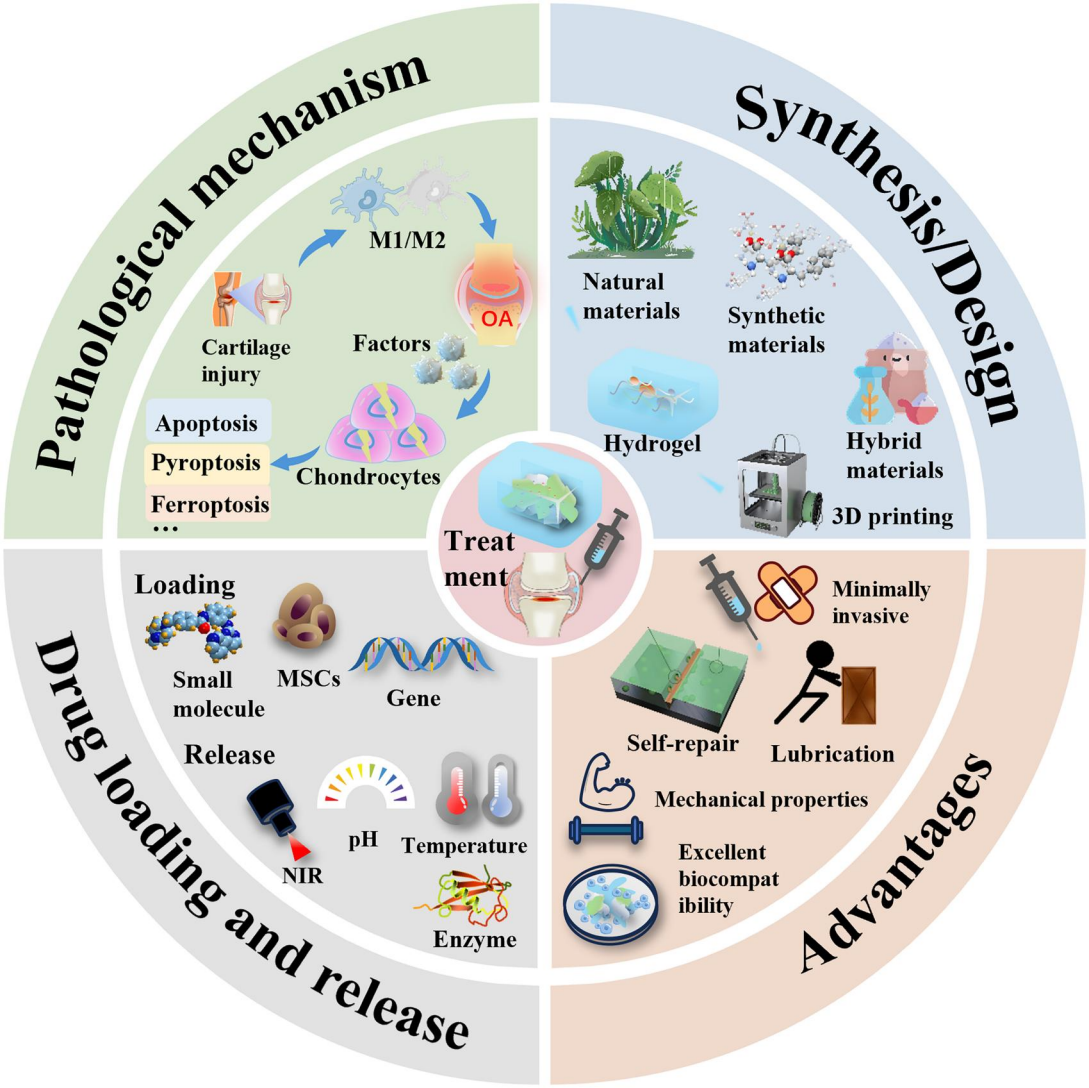
Schematic diagram of injectable hydrogels for cartilage repair based on OA mechanisms.

To achieve on-demand delivery of therapeutic agents and enable precise and effective regeneration of articular cartilage, this review systematically summarizes the pathological mechanisms following cartilage injury and current therapeutic limitations. It focuses on the design and application of injectable hydrogels as versatile platforms for delivering therapeutic substances. An optimal delivery platform should select appropriate therapeutics based on the pathological mechanisms of cartilage damage to promote efficient regeneration of both articular cartilage and subchondral bone (Figure 1). Overall, this review highlights the unique advantages of hydrogel strategies tailored to different therapeutic modalities offering valuable guidance for the advanced design of hydrogels in the field of cartilage repair.

## Articular cartilage structure and its pathological mechanisms of injury

### Joint cartilage structure

Articular cartilage is a specialized connective tissue that covers the ends of bones in diarthrodial joints, exhibiting a complex stratified architecture essential for its biomechanical functions, including load distribution, shock absorption and low-friction articulation. Proceeding from the articular surface toward the subchondral bone, cartilage is organized into four distinct histological zones. The superficial tangential zone consists of densely arranged flattened chondrocytes and collagen fibers oriented parallel to the surface, providing resistance against shear stresses and contributing to surface lubrication. Beneath this lies the transitional zone, where chondrocytes assume a more rounded morphology and collagen fibers adopt an oblique orientation, facilitating the gradual conversion of shear into compressive forces. The deep zone constitutes the majority of the cartilage volume, with chondrocytes organized in columnar arrays and collagen fibers aligned perpendicular to the articular surface, enabling effective resistance to compressive loads [19].

**Figure 2:**
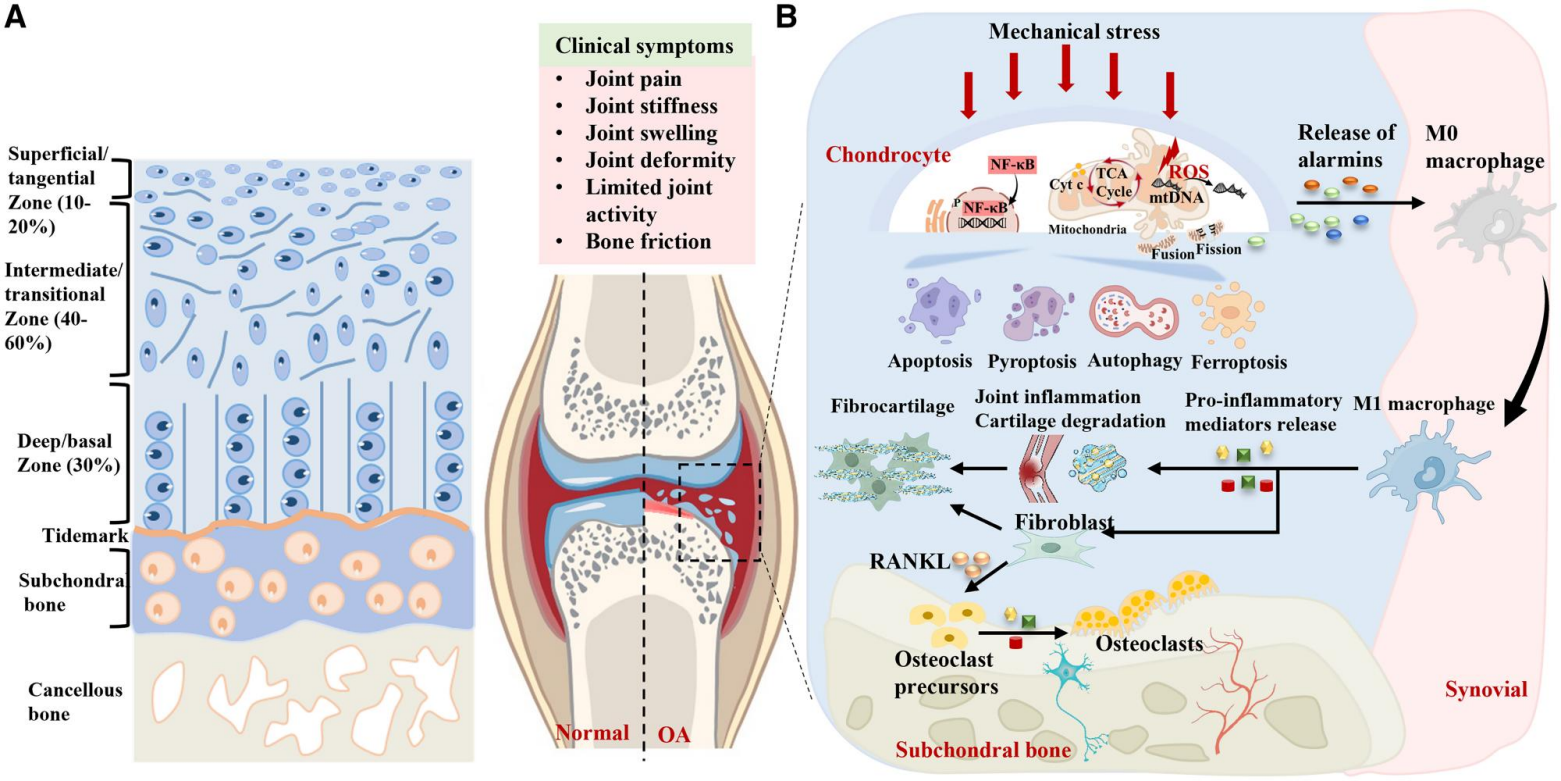
Schematic diagram of normal articular cartilage structure and molecular interaction mechanisms between cartilage, synovium and subchondral bone. (**A**) Normal articular cartilage structure. (**B**) Clinical manifestations of articular cartilage injury encompass pain, stiffness, swelling and deformity. This injury involves extensive chondrocyte death through processes such as apoptosis, pyroptosis, ferroptosis and autophagy. Released intracellular components subsequently recruit and activate inflammatory macrophages. These macrophages secrete inflammatory cytokines that drive the conversion of injured cartilage into fibrocartilage. Furthermore, the same cytokines activate specific signaling pathways to stimulate osteoclast proliferation. This activity results in exacerbated damage to the subchondral bone.

The deepest region of the cartilage is marked by a mineralized interface known as the calcified layer. This zone is characterized by a calcified ECM exhibiting substantially increased stiffness and is delineated from the overlying nonmineralized cartilage by a distinct basophilic boundary termed the tidemark. This interface serves as a critical mechanical transition that ensures stable integration between the compliant cartilage and the rigid subchondral bone. The calcified cartilage anchors directly into the subchondral bone plate, a dense cortical structure that provides structural support and dissipates mechanical loads into the underlying trabecular bone [19, 20]. The articular surface is bathed in synovial fluid, which is secreted by the synovial membrane and performs dual roles of nutrient delivery to the avascular cartilage and formation of a lubricating layer that minimizes friction during joint movement (Figure 2A).

### Cartilage inflammation–degradation coupling mechanism

The pathological process of OA initiates with chondrocyte dysfunction, which can be triggered by diverse factors including aging, genetic predisposition and mechanical stress. A central mechanism underpinning this dysfunction is mitochondrial impairment, characterized by depolarized membrane potential, reduced adenosine triphosphate (ATP) synthesis and excessive accumulation of reactive oxygen species (ROS), resulting in pronounced oxidative stress and bioenergetic deficit [21]. Imbalances in mitochondrial dynamics, such as excessive fission and inadequate fusion, coupled with impaired mitophagy, further exacerbate structural and functional mitochondrial deterioration [6]. The release of mitochondrial components into the cytosol or extracellular space accelerates chondrocyte death. Chondrocyte death in OA occurs through multiple distinct pathways, including apoptosis, pyroptosis, ferroptosis and autophagy-related mechanisms, rather than via a single modality [22–24]. These processes are activated by inflammatory or biomechanical stimuli, engage in crosstalk and act synergistically to promote irreversible chondrocyte loss and degradation of the ECM, thereby driving articular cartilage destruction.

Following chondrocyte death, cellular debris released into the synovial fluid is recognized as damage-associated molecular patterns (DAMPs) by the synovium, recruiting neutrophils and additional immune cells to the injury site [25]. Among these, M0 macrophages polarize toward a pro-inflammatory M1 phenotype upon recognition of mitochondrial or cytoplasmic DNA and secrete substantial quantities of inflammatory cytokines such as interleukin-1β (IL-1β) and tumor necrosis factor-α (TNF-α) [26]. These cytokines further disrupt chondrocyte metabolic homeostasis, activate the nuclear factor κ-B (NF-κB) signaling pathway and upregulate the expression of cyclooxygenase-2 (COX-2), inducible nitric oxide synthase (iNOS) and multiple matrix metalloproteinases (MMPs) [27]. These mediators promote catabolic breakdown of the ECM, suppress the synthesis of type II collagen and aggrecan and markedly amplify intra-articular inflammation (Figure 2B).

Progressive cartilage degeneration and joint space narrowing induce compensatory remodeling of the subchondral bone, characterized by sclerosis and osteophyte formation [26]. This remodeling process reflects an imbalance between osteoblastic bone formation and osteoclastic resorption, driven by dysregulated signaling pathways such as Wnt/β-catenin and RANKL/OPG that promote pathological bone remodeling [28, 29]. Concurrently, pro-inflammatory mediators including IL-1β and TNF-α diffuse from the degenerated cartilage into the subchondral compartment, where they stimulate aberrant angiogenesis and sensory nerve innervation, thereby accelerating trans-tissue disease progression [29]. Although initially adaptive, these structural and molecular alterations disrupt normal joint biomechanics, exacerbating stiffness and pain. Full-thickness cartilage loss results in bone-on-bone contact, where mechanical stress directly activates nociceptors and induces significant pain. Pain-related physical inactivity promotes periarticular muscle atrophy, further diminishing joint stability and initiating a self-perpetuating cycle of functional decline [30]. Beyond its mechanical role, the subchondral bone constitutes a dynamic biological niche populated by osteoblasts, osteoclasts, immune cells and vascular networks that engage in active molecular crosstalk with the overlying cartilage [31]. Pathological bone remodeling coupled with abnormal vascular invasion augments local inflammatory responses and promotes catabolic degradation of the cartilage matrix. These mechanisms collectively establish the central contribution of subchondral bone to osteoarthritis progression and provide a compelling rationale for multitarget therapeutic interventions concurrently addressing both osseous and cartilaginous compartments.

## Technical bottlenecks and limitations of OA treatment

### Limitations of pharmacological therapies

The clinical management of OA remains a major clinical challenge, largely due to the substantial limitations of current pharmacological strategies. First-line pharmacological agents, including nonsteroidal anti-inflammatory drugs (NSAIDs) and acetaminophen, primarily provide symptomatic relief by reducing pain and inflammation but fail to alter the underlying disease pathology. Prolonged administration of these drugs is frequently associated with adverse gastrointestinal, cardiovascular and renal effects, risks that are especially concerning in elderly populations [32]. Furthermore, the temporary symptomatic relief may mask ongoing joint damage, potentially leading to unintentional joint overuse and accelerated cartilage degradation, a phenomenon often termed analgesia-mediated hidden progression [33].

Among intra-articular therapies, corticosteroid and hyaluronic acid (HA) injections are commonly employed in clinical practice, yet both offer only transient and predominantly palliative benefits. Corticosteroids deliver rapid anti-inflammatory and analgesic effects, but repeated injections have been associated with cartilage toxicity and accelerated joint degeneration [34]. Although HA functions as a viscosupplement to improve joint lubrication and viscoelasticity, its clinical efficacy remains inconsistent, with considerable interpatient variability and a significant placebo effect complicating outcome assessment [35]. Importantly, neither therapeutic approach delays or halts structural disease progression, highlighting the critical need for disease-modifying treatments.

The progression of OA involves complex interactions across multiple pathological pathways, including inflammation, immune activation and intercellular communication, which collectively render single-target interventions largely ineffective in altering disease progression [36, 37]. Recent therapeutic approaches targeting key signaling pathways have shown promising outcomes. For example, metformin alleviates OA symptoms and retards structural deterioration by activating adenosine 5′-monophosphate-activated protein kinase (AMPK) and inhibiting mTORC1 signaling, thereby restoring the balance between autophagy and apoptosis [38]. Rapamycin, a mammalian target of rapamycin (mTOR) inhibitor, enhances chondrocyte autophagy and promotes joint homeostasis when delivered via intra-articular administration [39]. Additionally, pharmacological inhibition of the NOD-like receptor protein 3 (NLRP3) inflammasome suppresses the release of pro-inflammatory factors such as IL-1β and mitigates cartilage degradation in experimental models [40]. Similarly, inhibitors targeting ubiquitin-specific protease 7 (USP7) exhibit therapeutic potential by modulating ROS and NLRP3 activation [41]. Although these multipathway intervention strategies hold considerable translational promise, their clinical efficacy, optimal dosing and mechanistic consistency require further validation. Future drug development should advance beyond single-target agents by incorporating advanced nano-delivery systems for spatiotemporally controlled release and leveraging biomarker-based approaches to enable molecular subtyping and personalized OA treatment. Table 1 summarizes the mechanisms and limitations of investigated pharmacological and biologic agents for cartilage repair.

**Table 1:** Therapeutic molecular mechanisms for cartilage repair and their limitations.

Route	Typical molecule/drug	Primary indications	Mechanism	Results	Disadvantages	Ref.
Vein	CRISPR-Cas9, DOMADs, HDAC inhibitor	OA/Osteoporosis	OA genetics and epigenetics therapeutic option	Improving symptoms by affecting the expression of OA-related genes	Instability, nontargeting, poor bioavailability	[42, 43]
IP/Vein	LY294002, FGF18	OA	Inhibit PI3K/AKT/mTOR signal pathway	Reduce cartilage damage, decrease the degree of subchondral bone sclerosis and anti-inflammatory	Nontargeting, poor bioavailability, side effect	[44]
Oral	AG490, Acteoside	RA/OA	Inhibit JAK/STAT signal pathway	Inhibit the progression of OA and delay degradation	Low bioavailability, lack of targeting, risk of drug resistance	[45, 46]
Vein	Proteosome inhibitors, targeted IκBα ubiquination blockers	RA/OA	Block the NF-κB signaling	Reduce the inflammation response	Lack of targeting, toxic side effects	[47, 48]
Oral	NSAIDs (e.g., Ibuprofen, Naproxen, Celecoxib)	RA/OA	Inhibit cyclooxygenase enzymes, reducing prostaglandin production	Effectively delaying joint pain symptoms	Gastrointestinal toxicity, increased cardiovascular risk, renal impairment	[49]
Oral	Glucosamine & Chondroitin sulfate	OA	One of the components of cartilage, supporting its structural integrity	Promotes cartilage matrix synthesis, inhibits degradation and anti-inflammatory effects	Mild gastrointestinal side effects, slow onset of action	[50]
SubQ/IV	TNF-α Inhibitors (e.g., Adalimumab, Etanercept, Infliximab)	RA	Neutralize TNF-α, block inflammatory signals	Highly effective at reducing inflammation, halting radiographic progression in RA	High risk of infection, high risk of tuberculosis recurrence,	[51]
Oral	Acetaminophen/Paracetamol	Mild to moderate pain, fever	Central inhibition of prostaglandin synthesis	Effectively relieves pain associated with mild to moderate OA	Dose-dependent hepatotoxicity; ineffective against inflammation	[52]

### Defects in clinical surgical repair

**Figure 3:**
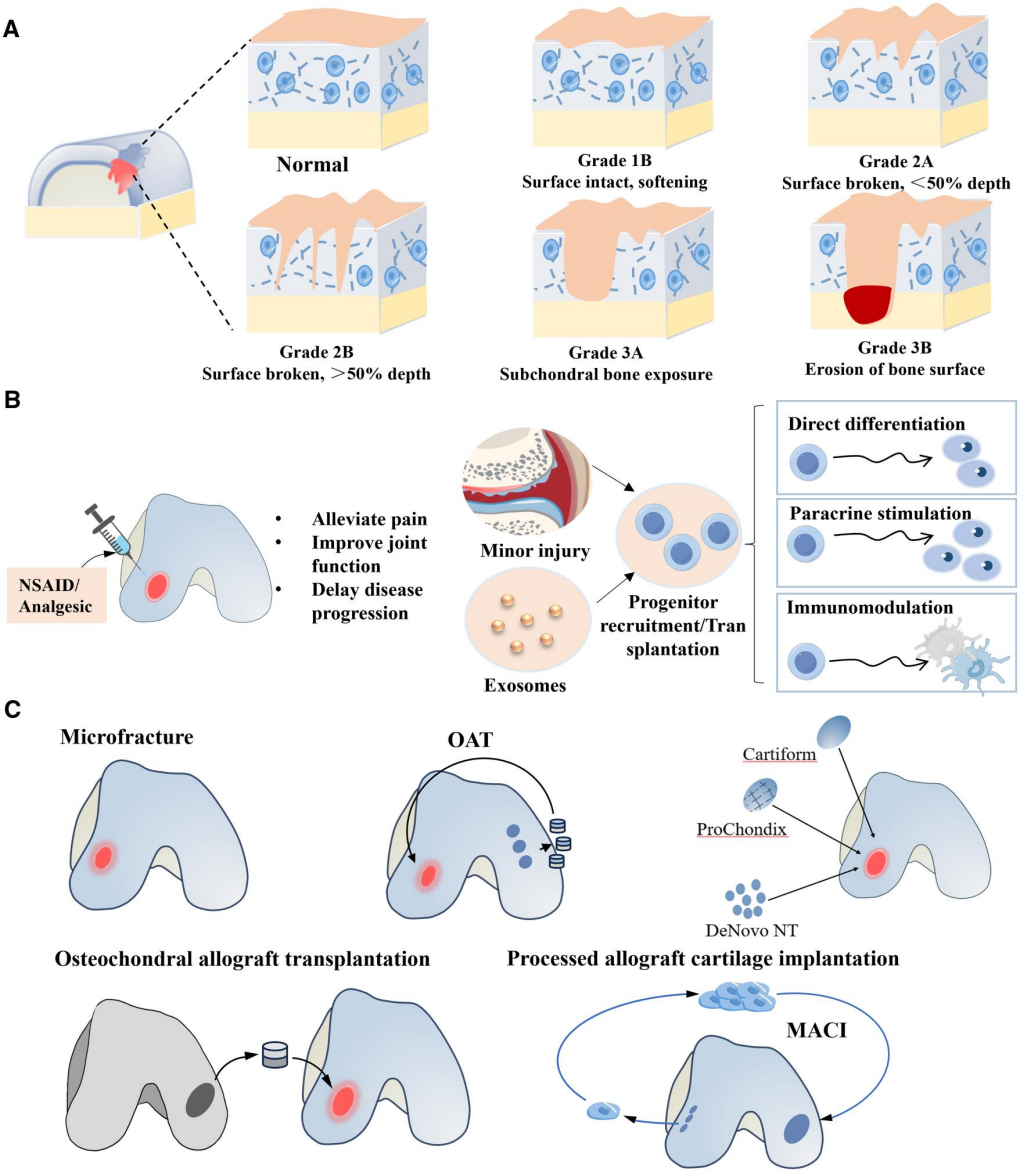
Grading of articular cartilage damage and current clinical treatment approaches. (**A**) Grading system for cartilage damage based on size and depth. (**B**) Mechanisms of drug or biological agent treatment for mild OA. (**C**) Surgeries commonly used for cartilage injury repair include microfracture, autologous cartilage bone grafting, allogeneic cartilage bone grafting, allogeneic cartilage implantation and autologous chondrocyte transplantation.

In OA, the cartilage surface exhibits structural alterations that share similarities with other forms of cartilage injury. According to established grading systems, cartilage lesions are categorized into five main stages based on lesion size and depth, with the repair strategy directly determined by the severity of the defect (Figure 3A). Mild cartilage injuries are commonly treated using conservative approaches, including load reduction, physical therapy and intra-articular injections such as anti-inflammatory drugs or platelet-rich plasma (PRP), to modulate the local biomechanical and biochemical environment. These interventions may be combined with specific biological agents to exert anti-inflammatory, analgesic and cell-proliferative effects (Figure 3B). In cases of moderate to severe cartilage damage, surgical interventions such as microfracture, autologous chondrocyte implantation or implantation of tissue-engineered biomaterial scaffolds are generally employed (Figure 3C).

While considerable advances have been made in repair techniques for small cartilage defects, significant limitations remain. Bone marrow stimulation techniques, which have evolved from open debridement to the Steadman microfracture approach, promote fibrocartilage repair by removing the calcified cartilage layer and recruiting mesenchymal stem cells from the subchondral bone. However, the resulting fibrocartilage, which is predominantly composed of type I collagen, exhibits a compressive strength of only 30–50% that of native hyaline cartilage and shows a degeneration rate of 68% at 5-year follow-up [7, 8]. To address these shortcomings, researchers have developed combinatorial strategies incorporating growth factors, collagen membranes or decellularized matrices. Despite these innovations, the current clinical evidence remains limited in quality and long-term therapeutic efficacy has yet to be fully validated.

Osteochondral autologous transplantation (OAT) enables anatomical reconstruction through the transfer of mature osteochondral units, allowing early weight-bearing and reducing rehabilitation time by approximately 40% compared to cell-based therapies [53]. However, this technique is limited by donor site availability and may lead to complications such as donor site lesions. Allogeneic osteochondral transplantation overcomes donor supply constraints but introduces challenges including immune rejection, risk of disease transmission and potential mechanical mismatch, contributing to a 10-year survival rate of only 66% [20]. Although cryopreservation or granulation of allografts can extend preservation time, these processing methods reduce chondrocyte viability by more than 60%, significantly compromising the quality of cartilage repair.

Cell-based repair techniques, such as autologous chondrocyte implantation (ACI) and matrix-induced autologous chondrocyte implantation (MACI), aim to regenerate hyaline-like cartilage [54, 55]. Notably, the two-stage surgical procedure increases infection risk, and *in vitro* expansion of chondrocytes often leads to dedifferentiation and loss of phenotypic stability, resulting in post-implantation cell survival rates below 40% [56, 57]. Although MACI shows high patient satisfaction at 5-year follow-up, its use is restricted to lesions larger than 3 cm^2^, with failure rates exceeding 55% in anatomically challenging regions such as the patellofemoral joint [57].

In summary, current treatment options remain largely confined to pharmacological symptom management, surgical correction of structural defects and passive biomaterial implantation. These interventions primarily act through isolated mechanisms and fail to reestablish a dynamically balanced ECM metabolism. This fundamental inadequacy highlights the urgent demand for multimodal integrated therapies capable of simultaneously modulating the inflammatory microenvironment, promoting endogenous ECM synthesis and supporting functional tissue regeneration.

## Hydrogel tissue engineering for cartilage repair

Hydrogel tissue engineering represents a multidisciplinary frontier integrating materials science, engineering, chemistry and biomedicine, with the goal of developing biomimetic biosubstitutes to restore the function of damaged tissues. By combining selected cell sources, tailored scaffold architectures and bioactive factors, this approach constructs dynamically tunable 3D microenvironments that are highly conducive to tissue regeneration [57–59]. Significant advances in biofabrication technologies, including 3D bioprinting, sophisticated decellularization techniques and mechanobiological insights, have markedly enhanced the clinical translation potential of hydrogel-based therapeutic strategies [60, 61]. Concurrently, the increasing prevalence of musculoskeletal disorders, particularly knee osteoarthritis in aging populations, coupled with continuous innovations in cartilage repair methodologies, is accelerating the development of the hydrogel-based engineered cartilage market.

**Figure 4:**
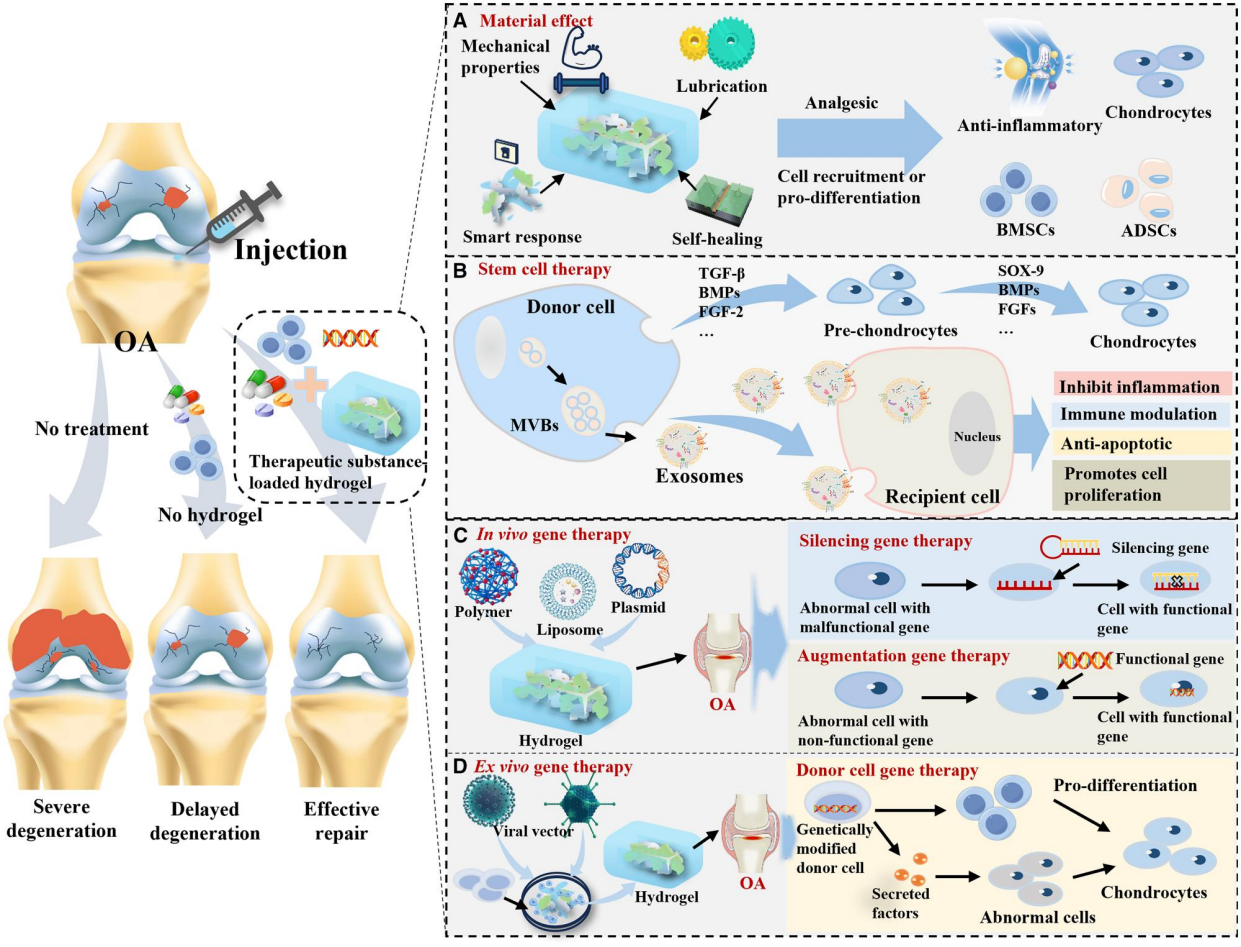
Schematic illustration of the therapeutic substance-loaded hydrogels in repairing cartilage damage. (**A**) Hydrogels possess inherent properties such as mechanical strength, self-healing capability, lubrication and smart responsiveness which can enhance cell viability and provide anti-inflammatory effects. (**B**) Stem cells promote cartilage repair primarily through differentiation into chondrocytes and paracrine activity. The latter mechanism involves the secretion of exosomes and other bioactive factors that orchestrate key therapeutic processes within the osteoarthritic joint. These processes include the suppression of inflammation, immunomodulation, inhibition of chondrocyte apoptosis and the promotion of endogenous cell proliferation. (**C**) Complementing this cellular approach, gene-activated hydrogels offer a sophisticated platform for localized therapy. These injectable systems function by encapsulating targeted nucleic acid carriers within a biocompatible hydrogel matrix. (**D**) Upon intra-articular injection, they provide a sustained and controlled release of genetic material directly to the diseased tissue.

Native cartilage tissue contains 70–80% water and exhibits characteristic viscoelastic mechanical properties, necessitating stringent biomimetic criteria for repair materials. Hydrogels have emerged as ideal scaffolds for cartilage regeneration due to their high-water content, tunable mechanical properties and excellent biocompatibility. Essentially consisting of 3D networks formed through physical or chemical crosslinking of natural or synthetic polymers, hydrogels can closely emulate the hydrated microenvironment of the native cartilage ECM. They are commonly categorized by crosslinking mechanism, material origin and functional features such as stimulus responsiveness or self-healing capability, leading to diverse hydrogel types [58, 62]. While extensive literature has thoroughly documented material selection, synthesis design and crosslinking strategies [63–65], this review will focus specifically on the design of hydrogels for articular cartilage repair. Injectable hydrogels offer distinct advantages for cartilage repair, characterized by four key attributes. Their sol-gel transition capability enables minimally invasive delivery of therapeutic agents directly to defect sites, minimizing secondary mechanical damage from bone-cartilage friction while improving patient comfort. These materials demonstrate tunable mechanical properties and exceptional shape adaptability, enabling effective filling of both extensive and irregular cartilage defects while preserving load-bearing capacity and biomimetic structural integrity [16]. Through incorporation of anti-inflammatory compounds or stem cell-recruiting molecules, functionalized hydrogels can modulate the local microenvironment to promote chondrogenesis and tissue regeneration. Furthermore, as versatile therapeutic reservoirs, hydrogels can be engineered for intelligent responsiveness to pathological cues, enabling precise spatiotemporal control over bioactive molecule loading and release for targeted treatment outcomes [66, 67]. These advantages originate from the inherent biomimetic properties of hydrogels that replicate native cartilage matrix characteristics, combined with their capacity to optimize therapeutic performance through enhanced cell viability maintenance, improved drug retention and facilitation of efficient paracrine signaling or transfection processes (Figure 4) [68, 69].

Current strategies for cartilage repair are undergoing a paradigm shift from static replacement toward dynamic intervention, centered on transforming hydrogels from passive fillers or carriers into intelligent therapeutic platforms capable of sensing and responding to disease signals. A key aspect of this evolution involves converting specific pathological features of the osteoarthritic microenvironment, including localized acidic pH, elevated ROS and overexpressed MMPs, into physical or chemical signals that trigger controlled therapeutic release [70, 71]. Through sophisticated molecular design, therapeutic agents such as small molecule drugs, growth factors, nucleic acids or cells can be conjugated to hydrogel networks using stimulus sensitive chemical bonds. These include acid labile hydrazone bonds, ROS cleavable boronic ester linkages or MMP degradable peptide sequences. Alternatively, therapeutic agents may be encapsulated within environmentally responsive micro or nanostructures. For instance, during the inflammatory phase, accumulated ROS in damaged cartilage can trigger degradation of boronic ester-containing hydrogel networks, leading to prioritized release of anti-inflammatory drugs to neutralize oxidative stress and modulate macrophage polarization [72, 73]. As inflammation subsides, the persistently acidic milieu or specific MMPs may further hydrolyze dynamic covalent bonds, enabling sequential release of chondrogenic growth factors or stem cell homing signals to guide subsequent tissue regeneration [11]. This stimulus-response-release cascade synchronizes therapeutic intervention with disease progression, significantly improving treatment precision while avoiding off-target effects and side effects associated with conventional drug delivery, thereby establishing a foundation for functional cartilage regeneration.

Beyond these classical pathological signals, emerging research aims to exploit more subtle pathological features within the cartilage damage microenvironment as advanced triggers for smart hydrogels. For example, during osteoarthritis progression, degradation of the cartilage ECM releases endogenous damage associated molecular patterns such as low molecular weight HA or oligomeric collagen fragments [74]. These molecules can exacerbate inflammatory cascades through receptor binding. In response, competitive hydrogel networks incorporating corresponding receptor ligands can be designed to undergo conformational changes or dissociation upon sensing specific matrix fragments, thereby actively releasing anti-inflammatory or anabolic factors to interrupt this vicious cycle [75]. Meanwhile, abnormally increased mechanical stress in diseased joints, though intangible, can be transduced into electrochemical signals via mechanosensitive ion channels or piezoelectric materials [76]. Embedding piezoelectric nanomaterials into hydrogels enables mechanical loading during joint movement to generate microcurrents. These microcurrents not only mimic the native electromechanical environment of cartilage, directly promoting chondrocyte proliferation and differentiation, but also act as switches for precise control of therapeutic molecule release [77, 78]. Furthermore, excessive pro-inflammatory cytokines produced by synovial tissue, along with aberrant nerve ingrowth and vascularization in later stages of injury, provide additional potential biomarkers for on demand hydrogel activation [79]. By integrating responsiveness to these diverse and time-dependent pathological signals, next-generation hydrogels will evolve beyond passive barrier functions into biomimetic systems capable of dynamically interpreting complex pathological language and delivering precise therapeutic responses, ultimately advancing from diagnostic intervention to synergistic repair in concert with the body’s own healing processes.

## Mechanical design of hydrogels for cartilage repair

Designing hydrogels with appropriate mechanical properties represents a significant challenge in cartilage tissue engineering. Native cartilage is a complex biphasic tissue characterized by a compressive modulus ranging approximately from 0.5–3 MPa and a tensile modulus that exhibits considerable anisotropy across its layered structure [80]. It also demonstrates exceptional stress-relaxation behavior and an extremely low friction coefficient. An ideal hydrogel scaffold should replicate these mechanical characteristics to provide a supportive microenvironment for cells and ensure seamless integration with surrounding native tissue [81, 82]. Key properties include matching stiffness, suitable viscoelasticity, effective lubrication and durability under cyclic loading. These attributes collectively determine whether the hydrogel can withstand intra-articular forces while maintaining compatibility with host cartilage.

Current strategies for enhancing mechanical performance often draw inspiration from natural cartilage architecture. By emulating the graded and layered organization of native tissue, researchers employ advanced fabrication techniques such as digital light processing-based 3D printing to create scaffolds with precisely controlled gradient structures [83]. These constructs typically feature a dense superficial region with small pores to resist mechanical stress, combined with a more porous interior to facilitate nutrient transport. This structural differentiation addresses both mechanical and biological requirements simultaneously. An alternative approach involves incorporating reinforcing fibers, such as polycaprolactone, within the hydrogel matrix. While the fibrous network bears the primary load, the hydrogel provides a bioactive environment, resulting in improved compressive strength and overall toughness [84].

Material composition and crosslinking strategies are crucial for enhancing the mechanical performance of hydrogels. Dual-network hydrogels, which integrate interpenetrating rigid and flexible polymer networks, effectively dissipate mechanical energy under load, achieving an optimal combination of high strength and wear resistance [85]. The addition of reinforcing phases such as graphene oxide or bacterial cellulose nanofibers can further enhance the modulus, strength and fracture toughness of the material [86]. Crosslinking methodologies have advanced toward hybrid systems that synergistically combine covalent bonds, ionic interactions and hydrogen bonding to balance mechanical robustness with dynamic adaptability [87]. The introduction of advanced dynamic chemistries further imparts self-healing capabilities, allowing structural recovery following damage.

Beyond serving as passive mechanical supports, hydrogels can be functionally engineered to actively engage with biological environments. A prominent example includes piezoelectric hydrogels, which generate electrical microcurrents in response to mechanical deformation during joint movement, thereby mimicking the native piezoelectric characteristics of cartilage and enhancing chondrocyte proliferation and differentiation [88]. Furthermore, hydrogels constructed from polyelectrolytes or modified with hydrophilic polymer brushes facilitate the formation of a stable hydration layer, significantly reducing friction and improving durability under articular loading conditions [89]. It is important to note that reported mechanical properties of cartilage exhibit considerable variation depending on measurement techniques, anatomical location and species. For example, the compressive modulus ranges from 0.2 to 1.5 MPa in rat cartilage and from 0.08 to 2.10 MPa in bovine cartilage [90, 91], reflecting both methodological and biological variability. In addition to matching mechanical performance, an ideal hydrogel should degrade at a rate synchronized with new tissue formation to ensure a smooth transfer of mechanical support to the regenerating cartilage. Strong interfacial integration with adjacent native tissue and subchondral bone is equally critical to prevent implant failure under long-term physiological loading.

## Hydrogel adhesion design for cartilage repair

In clinical applications for cartilage repair, the adhesive performance of injectable hydrogels is a critical determinant of therapeutic success. It governs the ability of the hydrogel to achieve stable retention and seamless integration with host cartilage within the dynamic joint environment, which is essential for delivering sustained mechanical support and facilitating biological repair [92]. Achieving effective adhesion extends beyond mere surface attachment and represents a multifaceted design challenge encompassing interfacial chemistry, dynamic mechanical interactions and biological integration. From a clinical surgical perspective, an ideal hydrogel must form immediate, strong and durable bonding with surrounding cartilage and subchondral bone upon injection to withstand continuous washing by synovial fluid, shear forces during cartilage loading and cyclic loads from early patient rehabilitation activities [93, 94]. If adhesion fails, any undesired displacement or loss of the hydrogel may not only lead to insufficient filling of the repair area but also generate loose bodies, potentially causing joint mechanical disorders or even inflammation. Therefore, design considerations must adopt a multidisciplinary approach, comprehensively integrating material science, biomechanics and practical clinical surgical needs to systematically optimize adhesion performance.

Specifically, the design strategy should shift from passive attachment to active integration. At the molecular level, bioinspired adhesive chemistry serves as the foundation. For example, modifying dopamine, catechol groups or introducing boronic acid moieties can form covalent bonds or strong dynamic bonds with amino and sulfhydryl groups in the cartilage matrix for establishing anchorage at wet interfaces [95, 96]. However, dependence on chemical bonding alone has been frequently inadequate. It is equally critical to engineer physical surface topographies that can mechanically interlock with the microstructure of the tissue. This can be accomplished by modulating the viscosity of the precursor solution and its gelation kinetics to enable controlled infiltration into tissue micropores or surface irregularities prior to solidification for forming complementary micromechanical interlocks [97, 98]. More importantly, enhancing adhesion must not come at the expense of any biological functionalities. Therefore, intelligent responsive elements should be incorporated, such as developing hydrogels with asymmetric adhesion properties—ensuring strong adhesion on one side to the defect area while maintaining a smooth surface on the other side to reduce friction with surrounding healthy cartilage. Ultimately, all designs must be validated via *in vitro* models simulating real joint environments and in large animal models, assessing adhesion stability under long-term mechanical stimulation and biodegradation. This ensures reliability at every step from the syringe to the defect site, achieving a qualitative leap from a mere filler to an integrated repair unit.

## Drug loading and release in hydrogels for cartilage repair

### Physical loading

The design of loading modalities and release kinetics in hydrogel-based drug delivery systems must be strategically aligned with the pathological alterations in the cartilage microenvironment to achieve optimal therapeutic efficacy. Following cartilage injury, the joint microenvironment undergoes significant biochemical changes including elevated oxidative stress, acidic pH and upregulated enzymatic activity, each of which provides unique opportunities for designing context-sensitive drug delivery approaches. Based on the intrinsic properties of therapeutic agents and the specific pathological features being targeted, drug loading strategies can be classified into two main categories: physical encapsulation and chemical conjugation, with the latter particularly suited to responding to pathological stimuli.

**Figure 5:**
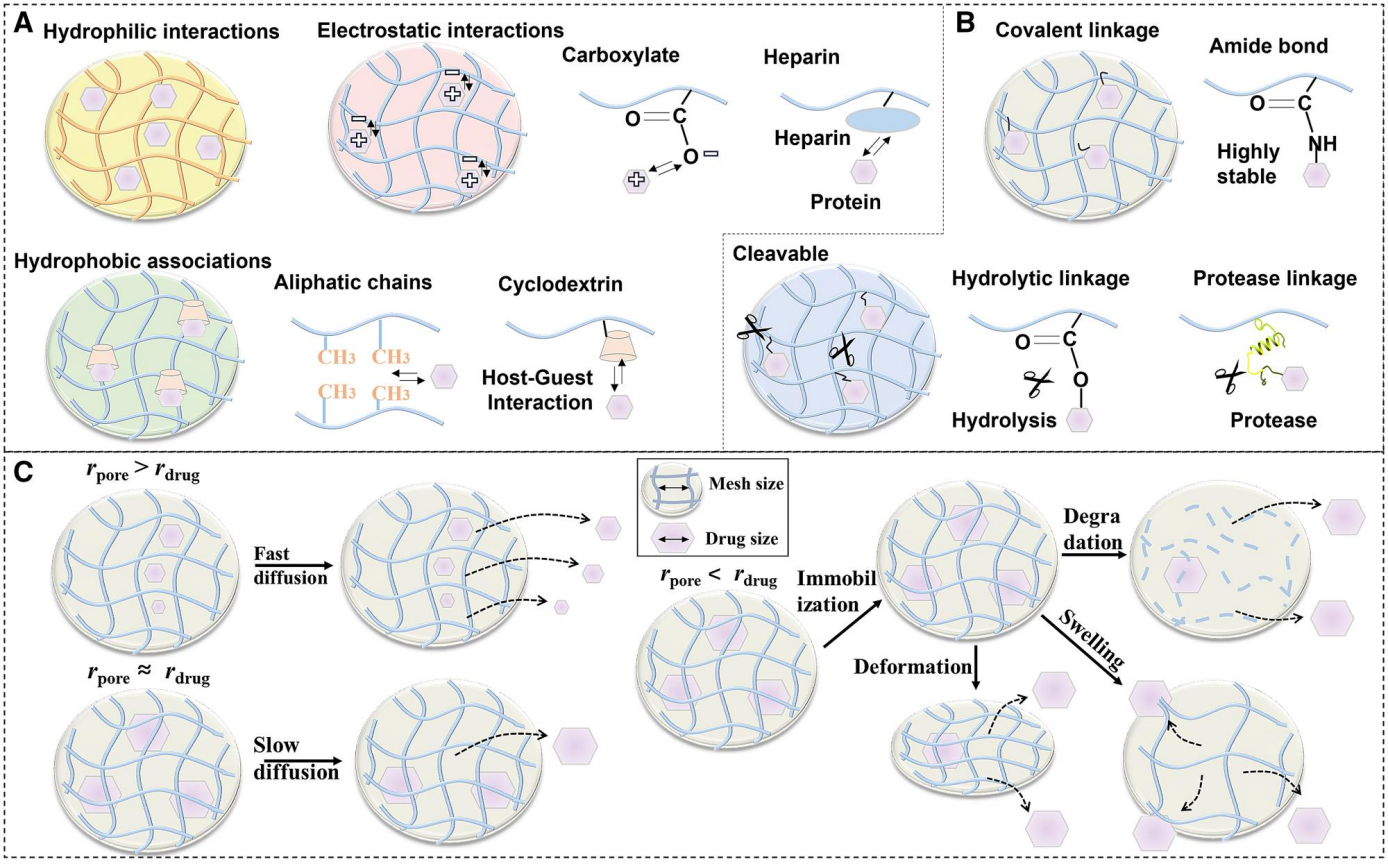
Main methods for drug loading into hydrogels. (**A**) Therapeutic cargo is loaded into hydrogels via physical interactions. (**B**) Therapeutic cargo is loaded into hydrogels via chemical modification. (**C**) The mechanism of hydrophilic molecules being released from hydrogels.

Physical entrapment primarily utilizes noncovalent interactions such as hydrophobic interactions, electrostatic forces and hydrogen bonding to adsorb and encapsulate therapeutic agents (Figure 5A). This approach demonstrates particular relevance to cartilage repair when designed with consideration for the altered electrostatic environment resulting from proteoglycan loss in damaged cartilage. For hydrophobic anti-inflammatory drugs such as curcuminoids, loading efficiency can be enhanced by creating hydrophobic microdomains within the hydrogel network [99]. Cyclodextrin-based supramolecular hydrogels represent an exemplary system that exploits amphiphilic architecture to selectively encapsulate hydrophobic molecules through host–guest interactions. Zhang *et al*. developed a dynamic hydrogel system based on coordination among metal ions, β-cyclodextrin (β-CD) and gelatin for cartilage repair [100]. The hydrophobic core of β-CD formed stable inclusion complexes with the chondroinductive factor kartogenin (KGN) through host–guest interactions, while the hydrophobic segments of the hydrogel further enhanced drug retention. This dual-interaction strategy significantly improved the encapsulation efficiency of hydrophobic drugs compared to conventional systems. Furthermore, it substantially enhanced drug retention within the joint cavity and promoted efficient repair of cartilage defects.

For hydrophilic therapeutic agents such as peptides and growth factors, conventional physical encapsulation often leads to premature diffusion due to weak matrix interactions [66]. This limitation becomes particularly problematic in cartilage repair where sustained presence of therapeutic molecules is essential for effective regeneration. To address this challenge, researchers have engineered electrostatic interactions that capitalize on the changing charge characteristics of damaged cartilage. Eric *et al*. [101] developed a composite hydrogel integrating HA/methylcellulose with poly(lactic-co-glycolic acid) nanoparticles (PLGA NPs), strategically modulating the zeta potential of PLGA surfaces to control electrostatic interactions with cationic therapeutics. When the negative surface charge density was optimized between −25 mV and −40 mV, the adsorption capacity for positively charged pituitary adenylate cyclase-activating polypeptide (PACAP) increased to 1.8 mg/g, representing a 2.3-fold enhancement over unmodified systems. This approach significantly prolonged PACAP retention within the cartilage microenvironment, directly addressing the challenge of maintaining therapeutic concentrations in cartilage repair applications.

### Chemical coupling

Chemical conjugation strategies represent a sophisticated approach for designing microenvironment-responsive drug delivery systems specifically tailored to the pathological features of osteoarthritic joints. By covalently attaching therapeutic agents to hydrogel matrices through enzymatically cleavable or hydrolytically labile bonds, this methodology enables precise spatiotemporal control over drug release in direct response to the biochemical alterations occurring in damaged cartilage (Figure 5B). The strategic selection of covalent bond types allows researchers to fine-tune both the stability of drug-carrier complexes and their responsiveness to specific pathological cues, thereby creating intelligent systems that synchronize therapeutic release with disease progression.

The acidic microenvironment that develops in inflamed articular cartilage provides a compelling rationale for employing pH-sensitive conjugation strategies. Dynamic covalent bonds, particularly ester and Schiff base linkages, have been extensively utilized in hydrogel-based delivery systems due to their tunable stability and pronounced environmental sensitivity [16]. Ester bonds demonstrate characteristic pH-dependent hydrolysis kinetics, with significantly accelerated cleavage under acidic conditions. This property has been strategically applied in cartilage repair through the development of a polyphosphazene-based hydrogel conjugated with anti-inflammatory drugs via carboxylic acid-derived ester linkages [102]. In this system, the ester bonds remain stable under physiological pH but rapidly hydrolyze in the acidic environment of inflamed cartilage [103], enabling targeted drug release precisely where pathological acidosis occurs. This pH-responsive behavior stems from protonation of the carbonyl oxygen under acidic conditions, which destabilizes the adjacent C-O bond and accelerates nucleophilic hydrolysis by water molecules.

Complementing ester-based systems, Schiff base bonds offer distinct advantages for drug delivery in the acidic microenvironments characteristic of progressive osteoarthritis. The dynamic equilibrium governing imine formation and hydrolysis permits accelerated drug release specifically under acidic conditions. This principle was effectively demonstrated in a poly(vinylamine)/PEG hydrogel system incorporating doxorubicin-PEG conjugates through Schiff base linkages [104]. While the imine bonds remained stable at neutral pH, protonation of the imine nitrogen in acidic environments substantially disrupted conjugate integrity, increasing the drug release rate by 4.8-fold compared to physiological conditions. This intelligent design minimizes premature drug leakage during circulation while ensuring enhanced delivery to acidic pathological sites within arthritic joints.

Beyond pH responsiveness, enzyme-cleavable conjugation strategies offer exceptional specificity for targeting the proteolytic environment of damaged cartilage. Enzyme-responsive peptide linkers represent a versatile approach for achieving pathology-mediated drug release, particularly in response to MMPs that are markedly upregulated in osteoarthritic joints. A compelling application of this strategy involves an MMP-responsive hydrogel fabricated through HA and gelatin crosslinking, designed to release KGN upon MMP-mediated cleavage [105, 106]. In the presence of MMPs overexpressed in degenerated cartilage, the peptide linkers undergo specific proteolysis, resulting in controlled KGN release that promotes chondrogenesis of resident stem cells. This system not only attenuates ECM degradation but also actively regenerates cartilage through timely growth factor delivery, demonstrating superior repair outcomes in preclinical models with significantly enhanced glycosaminoglycan deposition and improved histological scores compared to nonresponsive controls.

Furthermore, the elevated oxidative stress in osteoarthritic joints has inspired the development of ROS-responsive conjugation strategies. A notable example involves covalently linking therapeutic molecules to hydrogel scaffolds via thione bonds [107, 108]. These bonds remain stable under normal physiological conditions but undergo specific cleavage in the presence of excessive ROS found in inflamed synovium. This design enabled targeted anti-inflammatory therapy precisely at the site of oxidative damage, significantly reducing inflammatory mediators while protecting surrounding healthy tissue.

Beyond covalent conjugation and physical encapsulation, the strategic utilization of microstructural transformations in hydrogels provides an alternative pathway for drug delivery that aligns with the biomechanical demands of damaged cartilage. This approach leverages the inherent phase-transition behavior or structural rearrangement of hydrogel networks under specific pathological conditions to control therapeutic release. A particularly promising strategy involves designing shear-thinning hydrogels that undergo reversible structural changes in response to mechanical loading within the arthritic joint [109]. These systems capitalize on the abnormal biomechanical environment of osteoarthritic joints, where altered loading patterns and increased joint friction create unique opportunities for mechanically activated drug delivery. These injectable composite hydrogels can be engineered to temporarily disassemble their networks under mechanical stresses generated during normal joint movement. This structural transition enables the release of pre-loaded therapeutic molecules that stimulate chondrogenic differentiation of resident stem cells. As mechanical stress diminishes, the hydrogel network spontaneously reassembles, effectively terminating drug release and preventing unnecessary medication exposure. Such mechano-responsive platforms represent a sophisticated approach to drug delivery that harmonizes with the dynamic biomechanical environment of articular joints, offering spatially and temporally controlled therapeutic release precisely when and where mechanical demands are greatest.

### Drug release and stimuli-responsive regulation

The drug release behavior from hydrogels is governed by the synergistic interplay between the network architecture of the gel and external microenvironmental factors. A key regulatory mechanism involves diffusion kinetics, where the ratio of the hydrogel pore radius (*r*_pore_) to the drug molecule radius (*r*_drug_) determines the dominant release mechanism (Figure 5C). When *r*_pore_ > *r*_drug_, Fickian diffusion prevails, with larger pore sizes relative to the drug dimensions resulting in slower diffusion rates due to increased hydrodynamic resistance. When *r*_pore_ ≈ *r*_drug_, steric hindrance from polymer chains becomes significant and release efficiency can be optimized by modulating crosslinking density to adjust pore size. Notably, when *r*_pore_ < *r*_drug_, strong steric confinement effectively entraps drug molecules within the network, preventing release until matrix degradation occurs [110, 111]. This mechanism is particularly relevant for large therapeutic molecules or systems requiring extended drug retention.

The drug release profiles of hydrogels are not governed by a single universal mechanism but are highly dependent on their specific material characteristics. For example, certain highly concentrated hydrogels undergo substantial swelling upon hydration, while others tend to contract, leading to the extrusion of encapsulated therapeutic agents [16]. Hydrogels that operate through a swelling-mediated release mechanism generally possess a high density of hydrophilic functional groups, including amide, ether and hydroxyl moieties. When subjected to external stimuli such as variations in temperature, light, pH or enzymatic activity, these hydrogels exhibit strengthened electrostatic or hydrogen bonding interactions with water molecules [112]. This facilitates considerable water uptake into the polymer network, resulting in volumetric expansion and a significant rise in osmotic pressure, which collectively promote drug release. Through localized application or withdrawal of such external stimuli, the swelling and deswelling behavior of hydrogels can be reversibly modulated, allowing for spatially and temporally controlled drug delivery at the target site [71]. In contrast, hydrogels that function via a contraction-driven release mechanism are typically rich in hydrophobic constituents, such as long-chain alkyl, 3-block copolymers and poly(N-isopropyl acrylamide) [113]. Under changing environmental conditions, hydrophobic interactions and intramolecular hydrogen bonding become predominant, thereby impeding hydrogen bond formation between hydrophilic segments of the hydrogel and surrounding water molecules. This leads to matrix contraction and an associated extrusion effect, which accelerates drug release. Temperature-responsive hydrogels, such as those based on poly(N, N-diethylacrylamide), exploit a lower critical solution temperature to drive phase transitions [114, 115]. Below the lower critical solution temperature (LCST), the hydrogel remains hydrophilic and dissolved, while above this threshold, hydrophobic interactions induce gelation. Choi *et al*. [114] fabricated microcapsules with hydrogel membranes using an oil phase containing a copolymer of poly(N, N-diethylacrylamide) and benzophenone, whose capsule radius and membrane thickness exhibited temperature-dependent variations. The system achieved thermally regulated release of therapeutic molecules by reversibly adjusting the permeation cutoff threshold through temperature control as the swelling degree decreased with increasing temperature.

Hydrogel degradation constitutes another critical drug release pathway, serving as an alternative to diffusion-based mechanisms reliant on dynamic mesh size changes. This mode of release necessitates the incorporation of hydrolytically labile bonds within the hydrogel backbone, such as amide, ester, disulfide and boronic acid ester bonds. In systems where drugs are covalently tethered to the polymer network, release kinetics are primarily determined by the cleavage of these bonds and are therefore largely independent of hydrogel pore size. A major advantage of such configurations is their capacity to shield conjugated drug molecules, particularly peptide-based therapeutics, from rapid immune clearance. For example, Li *et al*. [116] developed novel hydrogel microspheres crosslinked with pH-responsive hydrazone bonds, which encapsulated liposomes functionalized with the cartilage-targeting peptide WYRGRL. Under acidic conditions, cleavage of the hydrazone bonds significantly enhanced liposome release compared to that observed under neutral conditions. These liposome-loaded hydrogel microspheres effectively promoted chondrogenesis through pH-triggered release and demonstrated anti-inflammatory effects by enhancing anabolic activities while reducing catabolic responses in chondrocytes. Although pH-responsive release strategies are theoretically promising, their clinical translation faces a major challenge regarding whether the extent and duration of pH fluctuations within the joint space are sufficient to trigger and sustain effective drug concentrations.

Moreover, while highly stable covalent bonds can retain drugs until matrix degradation occurs, selectively cleavable bonds can be engineered to rupture in response to specific biological cues or through predictable hydrolysis over time, enabling precise control over release timing. For example, Zheng *et al*. [11] engineered a natural hydrogel system crosslinked via β-CD and adamantane host–guest interactions, incorporating an MMP-3-cleavable peptide linker conjugated to a transforming growth factor-β1 (TGF-β1) mimetic peptide. Under pathological conditions characterized by elevated MMP-3 levels in injured cartilage, the peptide linker is selectively cleaved, enabling spatiotemporally controlled release of the therapeutic agent. This responsive delivery mechanism effectively downregulates the expression of catabolic and inflammatory mediators including MMP-3, IL-1β and COX-2, thereby suppressing inflammation, promoting hyaline cartilage regeneration and restoring glycosaminoglycan content toward physiological levels.

In summary, diverse drug release strategies can be achieved through physical encapsulation of therapeutic molecules, chemical conjugation and stimulus-responsive hydrogel design. These approaches allow for precise treatment tailored to the specific pathological features and progression stages of osteoarthritis.

## Hydrogel systems targeting inflammation and immunomodulation in articular cartilage

**Figure 6:**
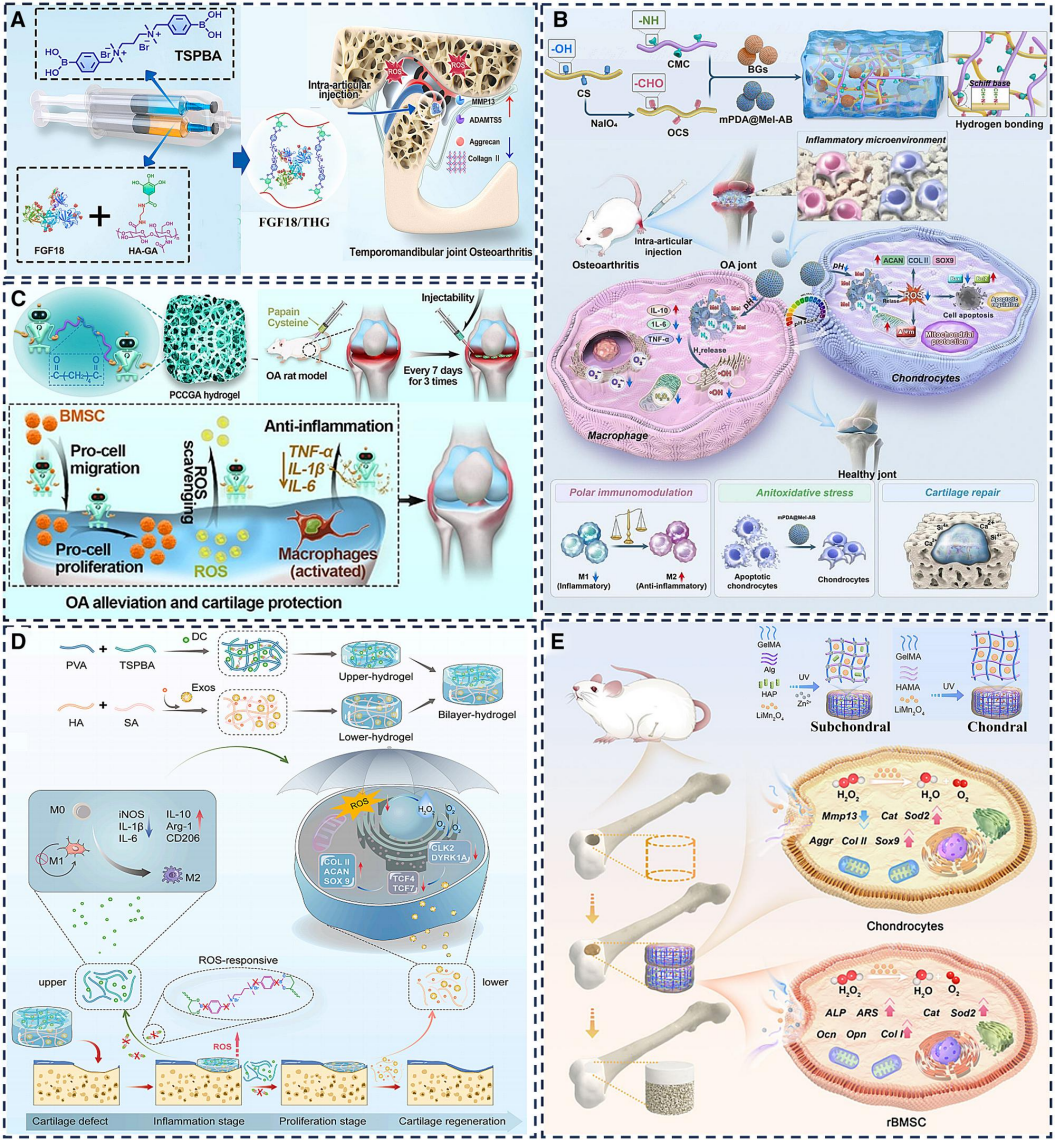
Application of hydrogel systems for joint cartilage inflammation and immunity. (**A**) Schematic illustration of ROS-sensitive hydrogel encapsulating FGF18 active protein treats TMJOA. Reproduced with permission from Ref. [118], Copyright (2025) Elsevier. (**B**) Schematic illustration of the preparation of mPDA@Mel-AB-loaded OCS/CMC hydrogel and its anti-inflammatory, antioxidant stress and chondrogenic effects. Reproduced with permission from Ref. [119], Copyright (2025) Elsevier. (**C**) Schematic illustration of the multifunctional PCCGA hydrogel exerting its effects in alleviating OA and protecting cartilage. Reproduced with permission from Ref. [120], Copyright (2023) Wiley. (**D**) Schematic illustration of a double-layer hydrogel promoting cartilage repair by modulating inflammatory responses through the release of exosomes and bioactive peptides. Reproduced with permission from Ref. [73], Copyright (2024) KeAi. (**E**) Schematic illustration of LiMn_2_O_4_-loaded bilayer hydrogel for simultaneous regeneration of cartilage and bone tissue. Reproduced with permission from Ref. [114], Copyright (2024) BioMed Central.

Hydrogel engineering strategies for inflammatory cartilage conditions are increasingly overcoming functional limitations through multidimensional innovation, reflecting a transition from simple designs to complex pathological interventions [3, 7, 117]. Temporomandibular joint OA (TMJOA), a degenerative condition characterized by inflammatory cartilage breakdown and subchondral bone erosion, currently lacks approved disease-modifying pharmaceuticals, with clinical management relying primarily on symptomatic treatment. To enhance the compromised bioactivity of fibroblast growth factor 18 (FGF18) in inflammatory environments, Kuang *et al*. [118] developed a ROS-responsive hydrogel crosslinked with N1-(4-boronobenzyl)-N3-(4-boronophenyl)-N1, N1, N3, N3-tetramethylpropane-1,3-diaminium (TSPBA) and gallic acid (GA)-modified HA. This hydrogel degrades under pathological conditions to scavenge ROS and enables the sustained release of encapsulated FGF18. It also mitigates the inflammatory suppression of FGF18’s reparative capacity, thereby inhibiting TMJOA progression (Figure 6A). While this ROS-sensitive approach improves growth factor functionality, it lacks the capacity for multidimensional regulation of the complex network of inflammatory mediators. Wang *et al*. [119] designed a composite hydrogel of oxidized chondroitin sulfate (OCS) and carboxymethyl chitosan (CMC) that incorporates mesoporous polydopamine nanoparticles (mPDA NPs) for drug delivery. This system allows for pH-responsive dual modulation. Melatonin released from the nanoparticles provides antioxidant effects, while ammonia borane generates hydrogen gas under acidic conditions to neutralize free radicals, together exhibiting potent ROS scavenging and anti-inflammatory activity. The addition of bioactive glass further enhances cartilage matrix repair by upregulating repair-related proteins (Figure 6B). The hydrogel’s hydration and hygroscopic properties help reduce intra-articular friction and inhibit osteophyte formation, thereby slowing osteoarthritis progression. This strategy moves beyond single-mechanism ROS regulation, yet challenges such as localized drug retention and suboptimal mechanical compatibility remain.

The evolution of hydrogel systems is increasingly focused on multifunctional integration to address the multifaceted complexity of joint inflammation. While polycitrate-based polymers possess inherent antioxidant and anti-inflammatory properties, their inability to self-polymerize has limited their use as sustained-release hydrogels. To address this, Wang *et al*. [120] developed a hydrogel solely from multifunctional polycitric acid-based (PCCGA) polymers via self-polymerization. This resulting PCCGA hydrogel is injectable, adhesive and features controllable porosity, elasticity and self-healing capabilities, making it suitable for OA alleviation and cartilage protection. It functions by scavenging intracellular ROS and downregulating pro-inflammatory cytokine expression, which promotes stem cell proliferation and exerts anti-inflammatory effects. Consequently, it helps restore uniform joint surfaces and cartilage ECM levels, effectively mitigating OA progression (Figure 6C). However, such homogeneous hydrogels possess uniform mechanical properties that can limit their adaptability across biologically heterogeneous tissue interfaces. In response, Lu *et al*. [73] designed a dual-layer hydrogel system for staged treatment following osteoarthritis injury. This system dually incorporates the anti-inflammatory drug sodium diclofenac (DC) and bone marrow mesenchymal stem cells (BMSCs). The upper layer, composed of phenylboronic acid-crosslinked polyvinyl alcohol, provides an initial rapid release of DC to suppress inflammation and promote macrophage reprogramming. The underlying HA layer facilitates the sustained release of exosomes to remodel the local microenvironment and enhance chondrogenic differentiation. This spatiotemporally controlled strategy enables seamless integration of inflammation suppression and tissue regeneration, overcoming functional constraints associated with single-component hydrogels through coordinated physicochemical and bioactive optimization (Figure 6D).

Advanced hydrogel designs further incorporate biomimetic layered structures to coordinate inflammation modulation with osteochondral regeneration. Hu *et al*. [121] developed a biomimetic bilayer hydrogel consisting of an upper cartilage layer made of HAMA, GelMA and LiMn_2_O_4_, alongside a subchondral bone layer formed from sodium alginate (Alg), GelMA, nano-hydroxyapatite (HAP) and LiMn_2_O_4_. The cartilage-like layer is functionalized with antioxidant nanozymes to protect against oxidative damage and support cartilage preservation, while the underlying bone-like layer exhibits osteoinductive properties that facilitate subchondral bone regeneration. This dual-functional design enables synchronous regeneration of both cartilage and bone, enhances biomechanical compatibility at the interface and thereby mitigates risks of interfacial failure (Figure 6E).

In summary, hydrogel engineering for cartilage inflammation treatment is undergoing a significant technological evolution, progressing from single ROS responsiveness to multimodal regulation, from structural mimicry to functional simulation and from passive drug release to active biological intervention. Through deeper integration of material design principles with pathological mechanisms, this field is shifting the therapeutic paradigm from symptomatic management toward genuine pathological reversal.

## Hydrogel systems for stem cell-based articular cartilage repair

### Adipose-derived stem cells

Adipose-derived stem cells (ADSCs), a principal subtype of mesenchymal stem cells isolated from adipose tissue, exhibit essential biological characteristics including self-renewal capacity, multipotent differentiation potential and low immunogenicity, rendering them an attractive candidate in regenerative medicine. In the context of cartilage repair, ADSCs can be efficiently directed toward chondrogenic differentiation via supplementation with specific growth factors or incorporation within 3D scaffolds, leading to the secretion of type II collagen and proteoglycans essential for functional cartilage formation. Recent advances in ADSC-laden hydrogel systems have progressed from simple single-material constructs to sophisticated platforms incorporating biomimetic architectures and spatiotemporally controlled multifactor release [122]. By integrating enhancements in mechanical compatibility, bioinstructive signaling and functional outcomes, these approaches are shifting the focus of cartilage repair from mere structural reconstruction to the restoration of biologically functional tissue, marking a significant evolution in regenerative therapeutic strategies [123].

**Figure 7:**
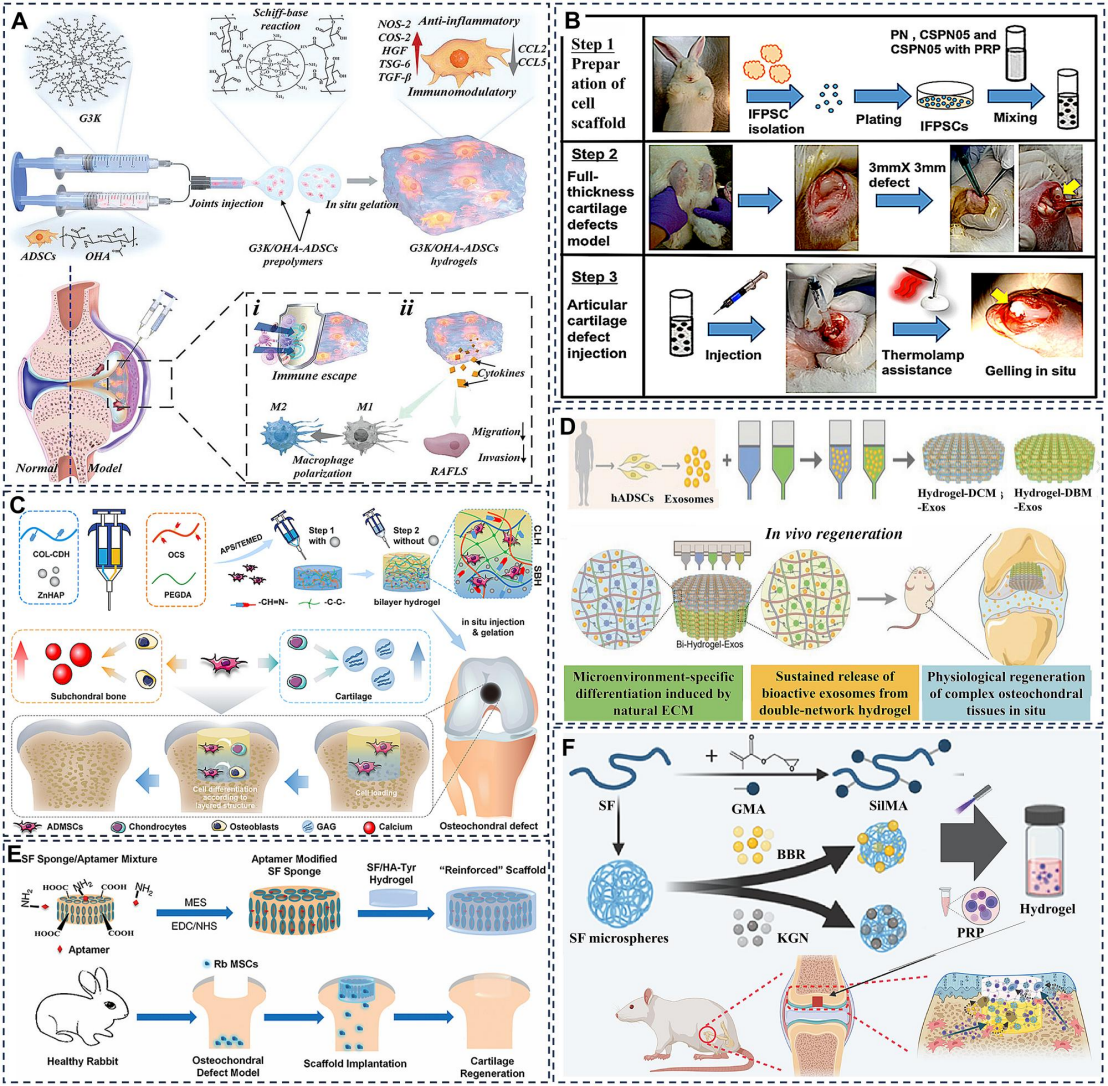
Application of stem cell-loaded hydrogel systems for articular cartilage repair. (**A**) Schematic illustration of the preparation of ADSCs-loaded dendritic poly-L-lysine and polysaccharide-crosslinked hydrogel and its mechanism of inhibiting cartilage inflammation via an immunomodulatory cellular communication network. Reproduced with permission from Ref. [124], Copyright (2023) Wiley. (**B**) Schematic diagram of surgical procedure using IFPSCs-loaded hydrogel for repairing full-thickness subchondral bone defects in rabbits. Reproduced with permission from Ref. [126], Copyright (2022) Elsevier. (**C**) Schematic illustration of the preparation of a double-layer hydrogel scaffold and its role in promoting cartilage repair via the activation of exogenous adipose-derived MSCs. Reproduced with permission from Ref. [127], Copyright (2023) Elsevier. (**D**) Schematic diagram illustrating the mechanism by which a bilayered 3D-printed biomimetic scaffold promotes cartilage regeneration in rats via exosomes secreted from hADSCs. Reproduced with permission from Ref. [128], Copyright (2023) Wiley. (**E**) Schematic diagram of MSCs-loaded silk fibroin/HA hydrogel scaffold preparation and its application in rabbit cartilage repair. Reproduced with permission from Ref. [129], Copyright (2019) SAGE Publications Inc. (**F**) Schematic diagram of a double-layer methacrylated silk fibroin hydrogel scaffold combined with PRP and silk fibroin–berberine microspheres for subchondral bone repair. Reproduced with permission from Ref. [130], Copyright (2022) Elsevier.

To enhance the survival and retention of transplanted ADSCs, innovative hydrogel designs have been developed with improved mechanical and biochemical properties. Zhu *et al*. [124] developed an innovative injectable hydrogel system composed of dendritic poly-l-lysine and functional polysaccharides for ADSC encapsulation and application in RA therapy. The hydrogel demonstrates notable self-healing characteristics, enhanced mechanical robustness and immunomodulatory capabilities. Upon implantation into rat cartilage defects, it facilitated the polarization of pro-inflammatory M1 macrophages toward the anti-inflammatory M2 phenotype and inhibited the migration of fibroblast-like synoviocytes, resulting in the effective amelioration of chronic inflammatory conditions (Figure 7A). Previous studies have established that infrapatellar fat pad-derived mesenchymal stem cells (IFPSCs) possess superior chondrogenic differentiation capacity compared to ADSCs, positioning them as a highly promising cell source for cartilage tissue engineering [125]. Capitalizing on this characteristic, Lin *et al*. developed a thermoresponsive chitosan-g-poly(N-isopropylacrylamide) (CSPN) hydrogel incorporating PRP to form a CSPN-PRP composite system. This construct demonstrates temperature-dependent injectability that satisfies minimally invasive clinical requirements while significantly enhancing chondrogenic/osteogenic gene expression and glycosaminoglycan deposition by IFPSCs in a rabbit model, resulting in substantially improved cartilage regeneration outcomes (Figure 7B) [126].

Bilayered hydrogel structures have been engineered to direct ADSCs toward region-specific differentiation for repairing both cartilage and subchondral bone. Cao *et al*. [127] developed a double-network hydrogel that mimics the native osteochondral interface through a dynamically crosslinked structure. The upper layer was loaded with ADSCs and a sustained-release system of insulin-like growth factor-1 (IGF-1), while the lower layer incorporated zinc-doped hydroxyapatite (Zn-HA). This dual-layer construct supported the differentiation of ADSCs into chondrocytes and osteoblasts within their respective layers, enhanced glycosaminoglycan secretion and promoted calcium deposition, thereby accelerating the regeneration of both cartilage and subchondral bone (Figure 7C).

Microenvironment-responsive hydrogels represent a promising strategy to maintain ADSC functionality under pathological conditions such as oxidative stress and acidic pH. Edirisinghe *et al*. [131] developed a drug-loaded dual-network hydrogel that enables sustained IGF-1 release for up to 21 days under injury-induced acidic and oxidative conditions through a combined pH- and ROS-responsive mechanism. This approach increased alkaline phosphatase (ALP) activity in ADSCs fourfold and promoted calcified nodule formation via activation of the bone morphogenetic protein (BMP)/Smad signaling pathway. However, such microenvironment-responsive hydrogels are often susceptible to rapid degradation, which can lead to premature stem cell release and increased vulnerability to hostile microenvironmental conditions. In contrast, 3D-printed bilayer scaffolds with microenvironment-specific design provide a superior protective barrier that enhances the survival and function of encapsulated cells. Li *et al*. [128] fabricated a bioinspired dual-network hydrogel scaffold via 3D printing, incorporating dECM and ADSC-derived exosomes to promote mesenchymal stem cell recruitment and *in vitro* cartilage formation. This microenvironment-tailored, heterogeneous bilayer scaffold further demonstrated the ability to accelerate simultaneous regeneration of cartilage and subchondral bone in a rat preclinical model (Figure 7D).

### Bone marrow mesenchymal stem cells

Bone marrow mesenchymal stem cells exhibit self-renewal capacity and multipotent differentiation potential, enabling them to differentiate into adipocytes, osteoblasts and chondrocytes. Notably, BMSCs display significant immunomodulatory and anti-inflammatory functions through paracrine mechanisms that facilitate tissue repair processes [132]. These properties render BMSCs promising candidates for OA treatment and attractive cell sources for cartilage tissue engineering. However, a major challenge in MSC-based cartilage regeneration is that these cells tend to undergo hypertrophic differentiation and exhibit limited efficiency in forming stable chondrocytes after induction. This hypertrophic shift promotes degradation of the cartilage ECM and leads to mineralization, ultimately resulting in structural calcification. Consequently, a key research objective involves developing strategies that guide MSCs toward stable chondrogenic phenotypes while preserving their differentiation capacity during *in vitro* culture.

Hydrogel systems provide a tunable 3D microenvironment that can effectively guide BMSCs toward stable chondrogenesis. Zhao *et al*. [133] addressed RA treatment challenges by developing a composite system consisting of a 3D-printed porous metal scaffold integrated with an infliximab-loaded hydrogel. This organic-inorganic hybrid system represented an early attempt to combine antirheumatic drug delivery with BMSC therapy, leading to significant reduction in inflammatory cytokines and enhanced subchondral bone regeneration in a rabbit RA model. However, the rigid metal scaffold provided limited mechanical compatibility for weight-bearing joint repair. To overcome this limitation, Wang *et al*. [129] developed a silk fibroin/HA (SF/HA) composite hydrogel in which SF, a natural polymer derived from Bombyx mori cocoons, provides mechanical strength and biocompatibility. The incorporation of HA enhances chondrogenic potential by activating CD44-mediated signaling pathways, thereby facilitating cartilage formation both *in vitro* and *in vivo* (Figure 7E). To further improve differentiation efficiency, Wang *et al*. [134] designed a photopolymerized double-network hydrogel that mimics the native ECM and coordinates with Fe³^+^ ions using poly(γ-glutamic acid). The incorporated Fe³^+^ ions not only enhance the mechanical properties of the hydrogel but also stimulate BMSC proliferation, upregulate cartilage-specific gene expression and promote the secretion of hydroxyproline and glycosaminoglycans.

**Figure 8:**
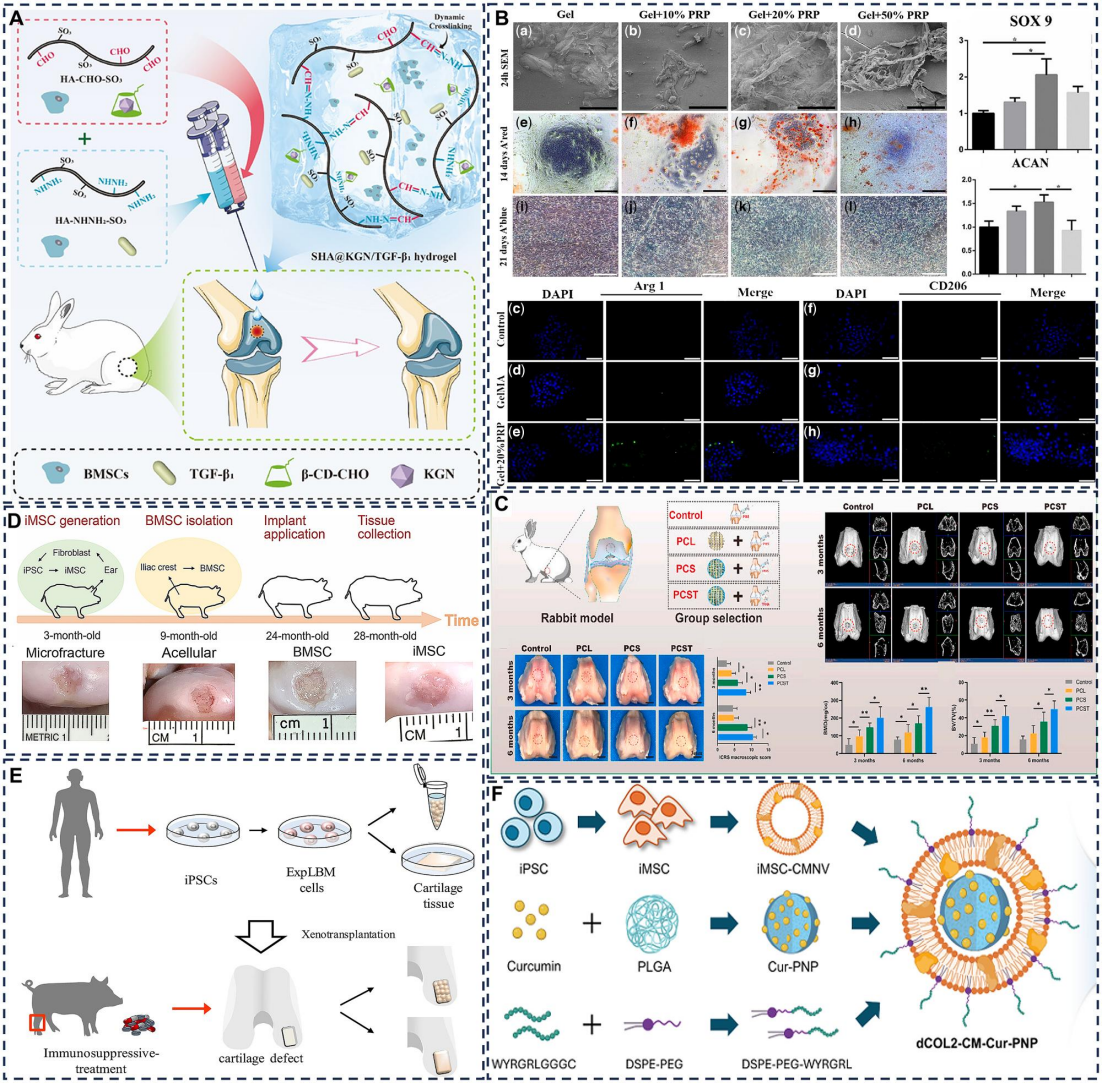
Application of stem cell-loaded hydrogels for OA repair. (**A**) Schematic illustration of the fabrication of a dual-drug-loaded HA-based hydrogel and its functional role in enhancing chondrogenic differentiation while inhibiting hypertrophy of BMSCs. Reproduced with permission from Ref. [135], Copyright (2023) Elsevier. (**B**) Representative morphological images of the 20% PRP-GelMA hydrogel demonstrating effective induction of chondrogenic differentiation in BMSCs and promotion of M2 macrophage polarization. Reproduced with permission from Ref. [136], Copyright (2021) Elsevier. (**C**) Macroscopic and micro-CT analysis of cartilage regeneration using MSC-loaded chitosan hydrogel/3D-printed PCL composite scaffolds. Reproduced with permission from Ref. [137], Copyright (2021) Elsevier. (**D**) Macroscopic evaluation of cartilage repair with autologous iPSC- and MSC-derived chondrocyte grafts in a miniature pig model. Reproduced with permission from Ref. [140], Copyright (2016) Elsevier. (**E**) Schematic diagram of decellularization treatment for hiPSCs differentiated into chondrocytes and their application in cartilage repair in a minipig model. Reproduced with permission from Ref. [144], Copyright (2025) Springer Nature. (**F**) Schematic illustration of the dCOL2-CM-Cur-PNPs hydrogel preparation. Reproduced with permission from Ref. [145], Copyright (2025) Elsevier.

Advanced hydrogel designs incorporating recruitment and differentiation cues can effectively guide BMSCs toward functional cartilage regeneration while suppressing undesirable hypertrophy. Jiang *et al*. [130] developed a bilayered methacrylated silk fibroin (SilMA) hydrogel fabricated through sequential photopolymerization to serve as a temporary ECM. The incorporation of PRP enhanced the early migration and pre-differentiation capacity of BMSCs, while the anchored silk microspheres loaded with KGN and berberine enabled sustained regulation of chondrogenic and osteogenic differentiation. This composite system effectively promoted the directed differentiation of recruited BMSCs even under inflammatory conditions, significantly enhancing the regeneration of both cartilage and subchondral bone (Figure 7F). However, a critical limitation following BMSC implantation remains their tendency toward hypertrophic differentiation. To address this, Gao *et al*. [135] designed an injectable dual-drug sulfated hyaluronic acid (SHA) hydrogel crosslinked through reversible Schiff base bonds, which co-encapsulates BMSCs with KGN and TGF-β1. The loaded TGF-β1 and KGN provided sustained-release kinetics for both drugs through electrostatic interactions and host–guest interactions, respectively (Figure 8A). The dual-drug-loaded hydrogel synergistically enhanced chondrogenic differentiation and suppressed BMSC hypertrophy, effectively promoting cartilage regeneration in both *in vitro* and *in vivo* settings.

Hydrogel-mediated immunomodulation represents a promising strategy to enhance BMSC-based cartilage repair by creating a favorable regenerative microenvironment. Utilizing digital micromirror device technology, Jiang *et al*. [136] fabricated a PRP–gelatin methacryloyl (GelMA) hydrogel scaffold to precisely modulate inflammatory responses and guide cellular differentiation. At an optimal concentration of 20%, PRP fine-tuned immune reactions while simultaneously enhancing BMSC chondrogenesis and promoting M2 macrophage polarization. In a rabbit model, this dual immunomodulatory and stem cell-regulatory approach improved subchondral bone regeneration quality by 40%, addressing a significant gap in material-mediated immune modulation (Figure 8B).

Combining hydrogels with supportive scaffolds and bioactive molecules can better replicate the native cartilage microenvironment for optimal BMSC chondrogenesis. Chitosan (CS) hydrogels exhibit outstanding biocompatibility and gene adsorption capacity, while their thermosensitive variants support clinical translation by facilitating nutrient exchange. 3D-printed poly(ε-caprolactone) (PCL) scaffolds offer mechanical stability, tunable degradability and controllable porosity. Li *et al*. [137] integrated these material advantages into a cartilage regeneration system based on a chitosan (CS) hydrogel/3D-printed PCL composite. This system, containing MSCs and recruited tetrahedral framework nucleic acids (TFNA), can be injected into the joint cavity. The 3D-printed PCL scaffold provided fundamental mechanical support, while TFNA created an optimal microenvironment for proliferation and chondrogenic differentiation of delivered SMSCs, thereby promoting cartilage regeneration and significantly improving cartilage defect repair outcomes (Figure 8C).

### Human embryonic stem cells

Human embryonic stem cells (hESCs) represent a highly promising cell source for cartilage regeneration due to their unlimited self-renewal capacity and multilineage differentiation potential. However, a major obstacle in this field involves establishing a stable and efficient culture microenvironment capable of reliably directing hESC differentiation toward specific chondrogenic lineages [138]. Early research demonstrated that combined bone morphogenetic protein 7 (BMP7) and TGF-β1 induction within HA hydrogels could generate homogeneous hyaline cartilage-like tissues from hESCs, although limitations persisted in differentiation efficiency and ECM secretion. To address this, chemically defined differentiation protocols utilizing specific matrix proteins and growth factor gradients under xeno-free conditions were developed, leading to efficient chondrogenic differentiation and significantly enhanced glycosaminoglycan (GAG) deposition [139]. Despite these advances, conventional two-dimensional culture systems fail to replicate the biomechanical gradients present in native cartilage, resulting in mechanical mismatch between regenerated and host tissues.

To overcome biomechanical incompatibility, 3D biomimetic scaffolds incorporating hESCs have emerged as a promising alternative. Microcavity hydrogel platforms successfully emulate the depth-dependent mechanical properties of cartilage through controlled porosity and stiffness gradients. In murine models, these platforms supported hESC-derived chondrocytes in forming native-like cartilage layers within eight weeks, demonstrating a 1.8-fold increase in type II collagen expression compared to conventional scaffolds without signs of calcification [140]. Further refinement involved integrating ROS-responsive NPs to enable dynamic mechanical regulation. These NPs facilitate sustained TGF-β3 release, which activates Smad2/3 signaling while simultaneously suppressing markers associated with hypertrophic differentiation.

### Induced pluripotent stem cells

The identification of a reliable cell source capable of restoring functional cartilage integrity represents a pivotal challenge in regenerative medicine. While bone marrow-derived mesenchymal stem cells can differentiate into chondrocytic lineages, their therapeutic efficacy is often compromised by donor age-dependent decline and inconsistent *in vivo* chondrogenic potential. Similarly, the derivation of functional chondrocytes from hESCs considerable ethical and technical limitations [141]. In contrast, induced pluripotent stem cells (iPSCs), first established in 2006, have transformed cartilage tissue engineering by providing an ethically uncomplicated and highly scalable alternative. These cells overcome the constraints of conventional cell sources through virtually unlimited self-renewal capacity and the ability to undergo directed differentiation into chondrocytes under precisely controlled conditions [142]. Nevertheless, the clinical translation of iPSC-based therapies continues to face several obstacles, including suboptimal graft survival rates and the necessity to refine differentiation protocols to minimize tumorigenic risks. Addressing these challenges through advanced biomaterial design and cytokine delivery systems is expected to improve the clinical viability of iPSC-mediated cartilage regeneration.

He *et al*. [140] utilized a microcavity alginate hydrogel platform to investigate 3D culture and chondrogenic differentiation of mouse iPSCs for generating scaffold-free cartilage grafts. The miPSC-seeded constructs formed cartilage microtissues exhibiting chondrocyte-like morphology with substantial type II collagen deposition, demonstrating the potential of 3D hydrogel systems to support iPSC-derived cartilage formation. This study represents the first successful differentiation of iPSCs into cartilage microtissues within a scaffold-free 3D environment. Nevertheless, applications of autologous iPSCs for articular cartilage repair in large animal models remain inadequately explored. To address this, Lee *et al*. [143] compared the therapeutic efficacy of autologous miniature pig iPSC-derived chondrocytes (iPSC-Ch) versus BMSC-derived chondrocytes (BMSC-Ch) for cartilage repair in a porcine femoral condyle model. The iPSC-Ch implants demonstrated superior regenerative outcomes, potentially attributable to increased CpG dinucleotide methylation in the COL10A1 promoter region, which suppresses hypertrophic differentiation and enhances ECM integrity (Figure 8D). These findings provide important translational evidence supporting the use of autologous iPSC-derived constructs for cartilage repair in large animal models.

To circumvent the risks associated with cell transplantation, iPSC-derived dECM has emerged as a promising cell-free therapeutic approach. This strategy involves differentiating human iPSCs into chondrocytes followed by decellularization; the resulting dECM significantly enhances chondrogenic differentiation of reprogrammed iPSCs through the GSK3β signaling pathway. In a minipig OA model, dECM implantation restored glycosaminoglycan deposition in defect sites to 89% of normal levels without teratoma formation risk. This approach not only addresses immunogenicity concerns but also utilizes endogenous ECM biomolecules to enable effective cross-species regulation, offering a versatile solution for clinical translation (Figure 8E) [144].

Concurrently, advanced drug delivery platforms incorporating iPSC-derived components are being developed to overcome limitations of conventional OA therapies. Traditional systems often suffer from rapid joint clearance and low biocompatibility, while OA-related inflammation exacerbates tissue damage and impedes regeneration. To address these challenges, the dCOL2-CM-Cur-PNPs system was developed using iPSC-derived mesenchymal stem cell membranes, curcumin-loaded NPs and damaged type II collagen-targeting phospholipids (Figure 8F). This platform enhances sustained release and cellular uptake of curcumin in OA chondrocytes, restores chondrogenic properties and modulates macrophage polarization by suppressing M1 activity while promoting M2 anti-inflammatory functions. Importantly, the specific binding of collagen II enables precise targeting of OA cartilage, as validated in both *in vitro* and *in vivo* models. In a DMM rat OA model, dCOL2-CM-Cur-PNPs effectively attenuated disease progression, demonstrating considerable potential as a next-generation OA-specific regenerative platform [145]. For a more complete summary, Table 2 summarizes recent studies on stem cell-laden hydrogels used for articular cartilage repair.

**Table 2:** Stem cell-loaded hydrogel for joint injury treatment.

Cell type	Hydrogel	Types of defects	Mechanism	Outcomes	Ref.
Pre-conditioned BMSCs	Methacrylated HA-Phenylboronic Acid (HAMA-PBA)	Rat OA model	Activates the PI3K-Akt signaling pathway to modulate macrophage polarization and inhibit osteoclast activation	Significantly reduced inflammation, promoted cartilage regeneration and improved joint function	[146]
BMSCs	Decellularized small intestinal submucosa hydrogel	Articular cartilage defect	Hydrogel modulates the local immune microenvironment; recruitment and chondrogenic differentiation of BMSCs	Cartilage-like tissue formed; significant increase in type II collagen expression	[147]
BMSCs	PEG-based hydrogel	Full-thickness cartilage defect	Hydrogel provides anti-inflammatory and antioxidant effects; promote BMSC migration and chondrogenic differentiation	Regenerated cartilage was continuous and smooth	[148]
ADSCs	EGCG and HA	Rat OA	EGCG scavenges ROS, protecting ADSC viability; hydrogel enables sustained release of TGF-β3 to induce chondrogenic differentiation	Induced synovial M2 macrophage polarization, promoted cartilage matrix formation	[149]
MSCs	HA-phenylboronic acid hydrogel (HA-TP)	Articular cartilage defect	Mimics the mesenchymal condensation process, inducing hypoxic metabolism and histone lactylation in MSCs, promoting organoid formation	Cartilage organoid formation efficiency increased 3-fold; type II collagen secretion increased	[150]
ADSCs	Piezoelectric poly(L-lactic acid) nanofiber hydrogel	Rabbit osteochondral defect	Piezoelectric effect activates TGF-β1 secretion by ADSCs; ultrasound triggers local electrical signals promoting cell migration and matrix deposition	Elastic modulus of neocartilage approached that of native cartilage.	[151]
Chondrocyte/MSC Co-culture	Functional peptide-based hydrogel	Articular cartilage injury	DFP/BFP peptides co-assemble into a porous structure; loaded TGF-β1 provides sustained release, promoting cell migration and signaling	Neocartilage thickness increased by 200%; GAG content increased 1.8-fold	[152]
iPSCs	Amniotic membrane hydrogel (AM)	Primate knee joint cartilage defect	AM hydrogel provides immunomodulation; co-delivered ADSCs synergistically promote PRG4 secretion	75% type II collagen coverage in the repair area; synovial inflammation reduced by 60%	[153]

In summary, these advancements represent a paradigm shift in regenerative medicine, transitioning from cell-based transplantation toward acellular ECM therapies and precisely targeted molecular delivery systems. This evolution addresses critical challenges in cartilage repair, including inadequate scaffold retention, mechanical deficiencies, immunological complications and hostile inflammatory microenvironments, thereby creating new opportunities for clinical translation.

## Hydrogel systems for gene-activated articular cartilage repair

The rationale for gene therapy in OA, which encompasses the use of microRNAs (miRNAs), small interfering RNAs (siRNAs), circular RNAs (circRNAs) and DNA plasmids, stems from its capacity to correct mutant gene sequences and target metabolically dysregulated proteins through effective gene transfer [154]. This approach is regarded as a promising supplement, and potentially a viable alternative, to current pharmacological treatments. Furthermore, impaired healing in OA patients, including deficient cartilage regeneration, has been associated with functional defects in MSCs and compromised proliferation and differentiation of osteoblasts. Genetically modified stem cells can enhance bone repair in OA by secreting therapeutic proteins and revitalizing dysfunctional endogenous cells. Successful gene therapy for OA requires the identification of specific mutant gene sequences involved in the disease process, followed by the selection of effective gene delivery vectors to ensure efficient transfection in both *in vitro* and *in vivo* settings [155]. Current strategies involve introducing plasmid DNA or RNA molecules such as miRNA, siRNA and circRNA into target cells to modify, activate or suppress functional genes, typically using nanoparticle-encapsulated gene fragments delivered via viral or nonviral vectors. Local administration of these vectors using injectable hydrogels has shown promise in reducing systemic side effects and improving transfection efficiency.

### MicroRNA

**Figure 9:**
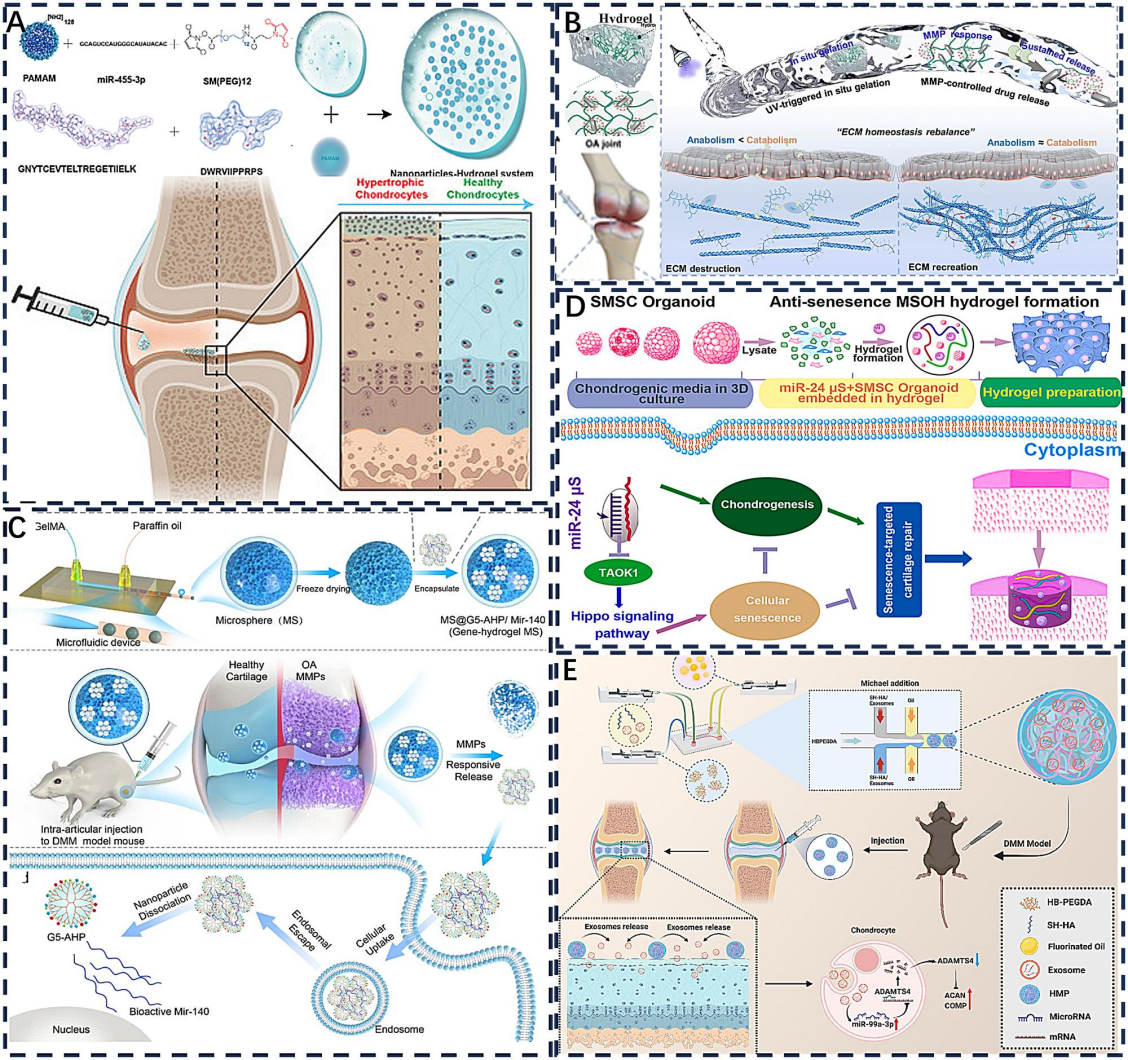
Application of gene-loaded hydrogel systems for articular cartilage repair. (**A**) Schematic illustration of PCEC hydrogel/CANC@miR-455-3p synthesis and its application in cartilage repair. Reproduced with permission from Ref. [157], Copyright (2025) Wiley. (**B**) Schematic illustration of MMP-responsive zinc oxide hydrogel regulating cartilage matrix homeostasis. Reproduced with permission from Ref. [158], Copyright (2025) Springer Nature. (**C**) Schematic illustration of the preparation of miR-140-loaded hydrogels and their therapeutic application for OA. Reproduced with permission from Ref. [159], Copyright (2025) Springer Nature. (**D**) Schematic illustration of miR-24 μS/SMSC organoid hydrogel enhancing chondrocyte homeostasis and cartilage defect repair. Reproduced with permission from Ref. [161], Copyright (2024) KeAi. (**E**) Schematic illustration of HMPs@Exos promoting OA repair via miR-99a-3b overexpression-mediated suppression of ADAMTS4 expression. Reproduced with permission from Ref. [162], Copyright (2023) Elsevier.

Exosome-based miRNA delivery systems overcome critical limitations such as short half-life and poor targeting efficiency through hydrogel encapsulation. Hu *et al*. [156] encapsulated human umbilical cord mesenchymal stem cell-derived small extracellular vesicles (hUC-MSC-sEVs) in a photo-crosslinked gelatin/nanoclay hydrogel, demonstrating that sustained release of miR-23a-3p suppressed the PTEN/AKT pathway. This intervention resulted in a 110% increase in chondrocyte proliferation and a 68% enhancement in type II collagen deposition. To address the short joint cavity half-life and off-target effects associated with free miRNAs, Long *et al*. [157] developed a cartilage-affinity nanocarrier (CANC) composed of 50% PEG-modified G5 polyamidoamine (PAMAM) dendrimers loaded with miR-455-3p. The system incorporated chondrocyte-targeting and endogenous peptides, and was embedded within a thermosensitive poly(caprolactone)-b-poly(ethylene glycol)-b-poly(caprolactone) (PCL-PEG-PCL) hydrogel. This design achieved full-thickness cartilage penetration in an OA model, reducing the OARSI score by 73% in miR-455-3p knockout mice while significantly improving targeting precision and retention time (Figure 9A).

Smart responsive hydrogels represent an advanced strategy to address the dynamic challenges of inflammatory and senescent microenvironments in cartilage repair by enabling temporally controlled release profiles that overcome the limitations of static delivery systems. Li *et al*. [158] created an MMP-responsive zinc oxide hydrogel that releases miR-17-5p when MMP-13 concentrations exceed 10 nM in the joint cavity. This system bidirectionally regulates ECM metabolism to restore cartilage homeostasis while featuring optimized rheological properties that reduce injection energy consumption by 40% and increase the support modulus threefold (Figure 9B). To achieve even greater release precision, Li *et al*. [159] employed microfluidic technology to produce monodisperse GelMA microspheres containing arginine–histidine–phenylalanine-modified G5 PAMAM/miR-140 NPs. This MMP-responsive hydrogel system enhanced type II collagen expression by 82%, suppressed a disintegrin and metalloproteinase with thrombospondin (ADAMTS) and MMP-13, and reduced osteophyte formation by 75% in a mouse OA model (Figure 9C).

The synergistic combination of material functionalization and metabolic reprogramming strategies improves the efficiency of cartilage regeneration, thereby facilitating its validation in large animal models. Zhu *et al*. [160] developed a self-healing hydrogel loaded with liposomes modified by DSPE-PEG-HA to deliver miR-140-5p, which regulates key modulators in cartilage regeneration. This novel hydrogel demonstrated effective release of miR-140-5p both *in vitro* and *in vivo*, exhibited excellent biocompatibility, and significantly increased the expression of the cartilage marker gene COL2A1, thus, promoting regenerative outcomes. Sun *et al*. [161] designed miR-24 microsphere/SMSC organoid hydrogels (MSOH) targeting thousand and one amino acid kinase 1 (TAOK1) to delay cellular senescence and support cartilage repair in osteoarthritis (Figure 9D). Their results showed that miR-24 delivered via the hydrogel reduced chondrocyte senescence and enhanced chondrogenesis in SMSC organoids by inhibiting TAOK1. Furthermore, the implanted MSOHs modulated chondrocyte homeostasis through regulation of cellular glycolysis and oxidative phosphorylation, which influenced cell cycle progression and ferroptosis, thereby attenuating joint degeneration and promoting cartilage formation. To achieve high transfection efficiency and sustained therapeutic action, Yin *et al*. [162] utilized microfluidic technology to fabricate injectable hydrogel microparticles containing ADSC-derived exosomes engineered with miR-99a-3p for osteoarthritis treatment. Their findings indicated that miR-99a-3p inhibits cartilage extracellular matrix degradation by targeting ADAMTS4, and the system displayed favorable sustained-release properties along with long-term therapeutic efficacy (Figure 9E).

### Small interfering RNA

**Figure 10:**
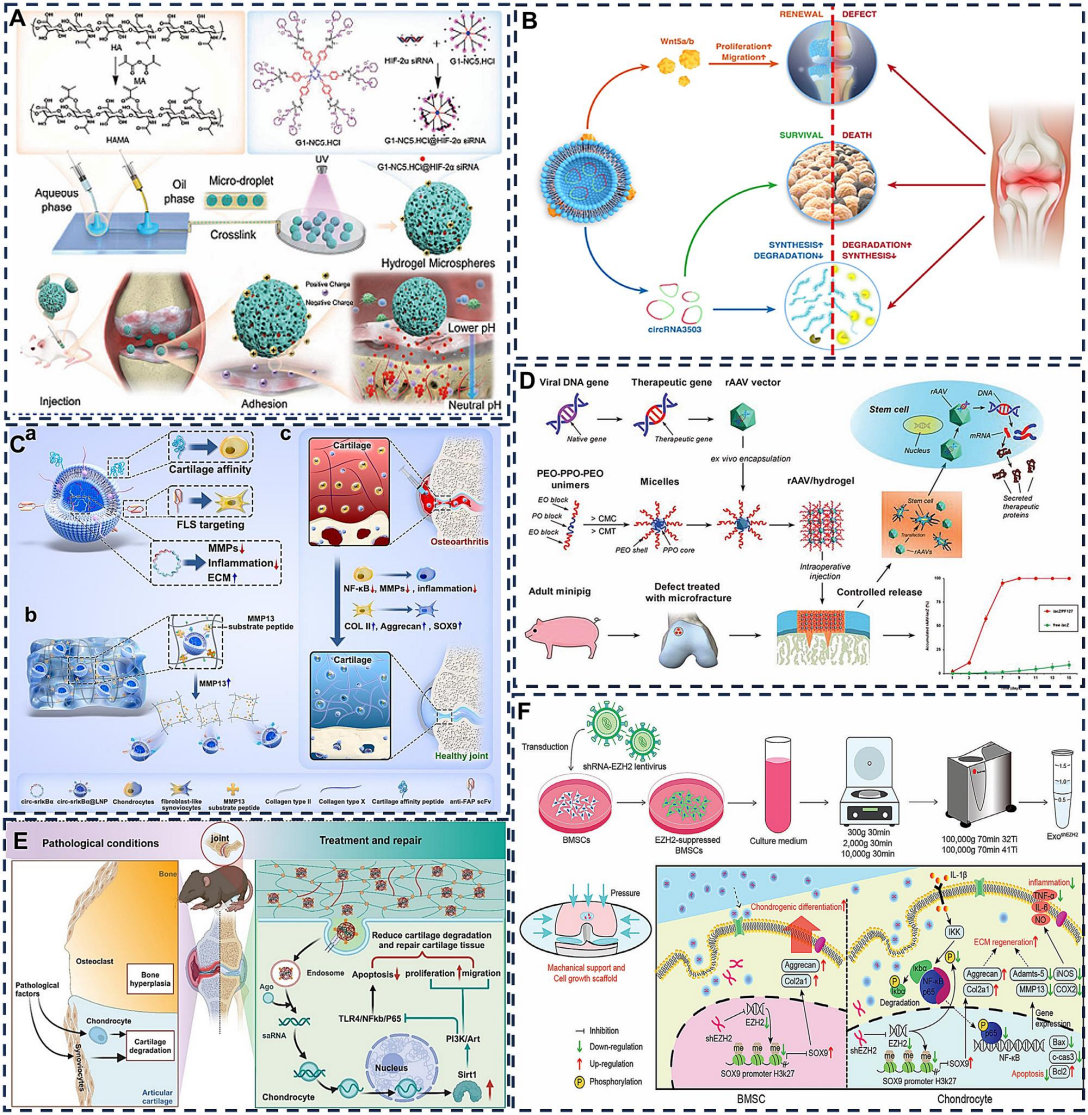
Application of gene-loaded hydrogels in cartilage injury repair research. (**A**) Schematic illustration of the preparation of HAMA/G1-NC5.HCl@siRNA hydrogel microspheres and their application for delivering therapeutic genes into the deep cartilage matrix in the treatment of OA. Reproduced with permission Ref. [165], Copyright (2023) Wiley. (**B**) Schematic illustration of circRNA3503-overexpressing small extracellular vesicles enhancing cartilage ECM synthesis and promoting cartilage regeneration. Reproduced with permission from Ref. [168], Copyright (2021) KeAi. (**C**) Schematic illustration of the MMP13-responsive hydrogel system (circsrIκBα@LNP-SHC) for treating OA. Reproduced with permission from Ref. [169], Copyright (2025) Elsevier. (**D**) Schematic diagram of a recombinant adeno-associated virus/hydrogel system employed for repairing full-thickness cartilage defects in the knee joint of a minipig. Reproduced with permission from Ref. [170], Copyright (2020) Wiley. (**E**) Schematic diagram of the therapeutic mechanism of the OCCG-4LF@saRNA hydrogel in treating OA. Reproduced with permission from Ref. [171], Copyright (2025) Elsevier. (**F**) Schematic diagram of shRNA-EZH2 lentiviral transduction and ExoshEZH2 extraction, along with the mechanism of EZH2 gene downregulation. Reproduced with permission from Ref. [172], Copyright (2025) Wiley.

Research on RNA-loaded hydrogels presents innovative therapeutic strategies for OA by combining the precise regulatory function of siRNA with the efficient delivery capabilities of hydrogel systems. As a core component of RNA interference technology, siRNA specifically binds to complementary mRNA sequences, inducing target mRNA degradation and effectively silencing genes involved in cartilage degradation. This process helps inhibit inflammatory responses and reduce ECM breakdown. However, the clinical translation of siRNA faces several challenges, including susceptibility to enzymatic degradation by RNases, lack of tissue-specific targeting and the requirement for sustained local release within the joint space to maintain therapeutic efficacy [163, 164]. To address these issues, Chen *et al*. [165] developed a pH-responsive phosphazene dendrimer-based nanocarrier (G1-NC5.HCl@siRNA) loaded with HIF-2α siRNA, which was encapsulated into HA microspheres using microfluidic technology. This system enables targeted intra-articular delivery and exhibits enhanced penetration and sustained release within the degenerative cartilage microenvironment at pH 6.6, significantly suppressing MMP-13 expression while promoting collagen II regeneration (Figure 10A). Dysregulated inflammatory cytokine levels and disrupted immunometabolism within the joint microenvironment are critical drivers of RA progression. To address these pathological mechanisms, Song *et al*. [166] developed an alginate hydrogel crosslinked with Zn^2+^ ions that encapsulates TNF-α siRNA within hyperbranched poly(β-amino ester) NPs. This system undergoes gradual degradation under acidic conditions, releasing TNF-α siRNA to modulate M1/M2 macrophage polarization, suppress inflammation, enhance fatty acid oxidation and promote cartilage repair.

In addition to inflammatory factors, proteases such as MMP-13 serve as important therapeutic targets for cartilage regeneration. Wang *et al*. [167] developed a novel approach by complexing MMP-13 siRNA with polyethyleneimine-polyethylene glycol-modified Fe_3_O_4_ NPs to create siRNA-loaded nanocarriers. These were incorporated into a HA hydrogel crosslinked with poly(vinyl alcohol) and modified with phenylboronic acid, resulting in a dual-functional system exhibiting both ROS responsiveness and RNA interference capability. When administered via intra-articular injection in a mouse OA model, this hydrogel significantly reduced cartilage matrix degradation, alleviated synovitis and subchondral bone sclerosis, delayed disease progression and improved pain-related outcomes.

### Circular RNA

Circular RNAs exhibit enhanced stability and resistance to enzymatic degradation compared to linear RNAs due to their covalently closed-loop structures, positioning them as promising therapeutic agents for sustained chondroprotective therapy. The integration of circRNA with hydrogel-based delivery systems capitalizes on the innate biostability of the former and the sustained release profile of the latter, presenting a highly promising strategy for long-term gene regulation in the treatment of arthritis. Melatonin-induced circRNA3503 produces therapeutic effects by competitively binding to miR-181c-3p and let-7b-3p, thereby reducing IL-1β-induced chondrocyte apoptosis *in vitro* [168]. However, the translational potential of free circRNA formulations is constrained by inadequate targeted delivery and limited retention at pathological sites. To address these challenges, Tao *et al*. [168] developed a composite system combining circRNA3503-overexpressing synovial mesenchymal stem cell-derived exosomes with a thermosensitive PCL-PEG-PCL hydrogel. This platform enabled sustained circRNA3503 release over 28 days in OA models, resulting in a 55% reduction in ECM-degrading enzymes and significant mitigation of cartilage degeneration (Figure 10B).

To achieve on-demand circRNA release, Li *et al*. [169] designed an MMP-responsive smart hydrogel system by encapsulating a circRNA encoding the NF-κB suppressor srIκBα into lipid NPs and embedding them within a silk fibroin hydrogel crosslinked with MMP-sensitive peptides (circ-srIκBα@LNP-SHC). Upon exposure to MMP concentrations exceeding 15 ng/ml in the joint cavity, the hydrogel degraded and released the lipid NPs, yielding a 3.8-fold improvement in chondrocyte targeting efficiency compared to conventional delivery systems (Figure 10C). This dual-responsive system reduced TNF-α and IL-1β levels by 71% in a rat OA model and restored type II collagen production to 89% of normal levels in *ex vivo* human cartilage experiments.

### Plasmid DNA and other

Plasmid DNA therapy seeks to enhance functional protein expression through the delivery of genes encoding therapeutic proteins; however, its clinical translation has been hindered by low transfection efficiency and transient gene expression. To overcome these limitations, hydrogel-mediated gene delivery systems have been engineered to provide spatiotemporally controlled release of genetic material, thereby minimizing vector dispersion and improving localized transfection. For instance, a thermosensitive poly(ethylene oxide)-poly(propylene oxide)-poly(ethylene oxide) (PEO-PPO-PEO) triblock copolymer hydrogel enabled sustained release of a recombinant adeno-associated virus carrying the SOX9 gene in a minipig cartilage defect model [170]. This approach significantly enhanced collagen fiber organization and conferred protective effects on the subchondral bone (Figure 10D). Despite these advances, concerns regarding the immunogenicity of viral vectors have prompted exploration of nonviral alternatives.

In response to the limitations of viral vectors, nonviral strategies such as small activating RNA (saRNA) have garnered increasing interest. Xu *et al*. developed a polylysine-modified ferritin vector capable of lysosomal escape via the proton sponge effect, allowing pH-responsive saRNA release. Loaded with Sirt1-saRNA, this vector was incorporated into an injectable, thermosensitive and self-healing hydrogel composed of chitosan, oxidized chondroitin sulfate and β-glycerophosphate, forming the OCCG-4LF@saRNA system [171]. Experimental results demonstrated that this system significantly upregulates Sirt1 expression in chondrocytes, suppresses apoptosis and attenuates OA progression (Figure 10E).

To address the efficacy limitations of single-gene regulation, novel composite hydrogels integrating epigenetic modulation with exosome therapy have been developed. The ExoshEZH2@HG photo-crosslinkable hydrogel consists of modified HA, gelatin and EZH2-shRNA-engineered exosomes, offering favorable mechanical properties and sustained release kinetics [172]. In a rat model, this platform facilitated cartilage repair through a dual mechanism: exosome-delivered shRNA inhibited EZH2 expression, thereby suppressing the NF-κB inflammatory pathway and reducing histone hypermethylation, while simultaneously promoting BMSC migration, differentiation and ECM regeneration (Figure 10F). This combined action resulted in the formation of hyaline cartilage-like tissue, demonstrating that multitargeted synergistic strategies can overcome the limitations of single-gene carriers in terms of anti-inflammatory efficacy and cellular recruitment.

In summary, gene-loaded hydrogels offer multifaceted therapeutic benefits for cartilage regeneration by enabling distinct nucleic acid-based regulatory strategies. These systems achieve precise modulation of complex genetic networks through miRNA delivery, facilitate efficient and sustained silencing of disease-associated genes using siRNA and exploit the inherent stability of circRNA integrated with controlled-release platforms for prolonged activity. Furthermore, plasmid DNA incorporation allows the joint space to function as a localized and sustained therapeutic protein production site. Together, these approaches highlight the unique capacity of hydrogels to protect genetic cargo, provide spatiotemporal release control and synergistically convert diverse nucleic acid therapeutics into a coordinated, long-term regenerative process directly within the defect microenvironment.

## Prospects and conclusions

The core point of a traditional injectable hydrogel strategy is the leverage of a hydrogel as an instructive, temporary ECM. The mechanism of action relies on the hydrogels’ biocompatibility, mechanical support and in-situ delivery of loaded bioactive molecules to create a microenvironment at the defect site for tissue regeneration. This environment recruits and guides endogenous host cells to facilitate repair. For example, smart responsive hydrogels can react to temperature, pH or mechanical stimuli within the joint to achieve controlled release of drugs or growth factors. The advantages of this approach include a relatively direct clinical translation pathway, suitability for minimally invasive surgery due to injectability and high flexibility and controllability in material design and functionalization.

In contrast, the cartilage organoid method represents a more advanced replacement strategy, the fundamental mechanism of which involves using stem cells or chondrocytes cultured *in vitro* within a carefully designed biomaterial support system to pre-form living tissue modules [173]. These modules possess a 3D structure, cellular composition and functional properties that closely mimic native cartilages. For example, Su *et al*. [174] utilized a digital light processing system to bioprint BMSCs within a DNA-silk fibroin hydrogel slow-release system, constructing millimeter-scale cartilage organoids. The organoids demonstrating the optimal chondrogenic phenotype were then selected for repairing articular cartilage defects in a rat model. These cartilage organoids exhibited higher expression of cartilage markers and the regenerated tissue displayed a gene expression profile similar to healthy cartilage four weeks post implantation. Beyond serving as sophisticated implants, a unique and powerful advantage of organoids is their role as exceptional research models. They can establish highly realistic models of osteoarthritis pathology and be employed for high-throughput drug screening, such as the use of fluorescently labeled cartilage organoids to identify drug targets that promote cartilage formation while inhibiting hypertrophy [175, 176].

The successful clinical translation of hydrogel-based therapies for cartilage repair faces multifaceted challenges that can only be addressed through close collaboration among material scientists, pharmaceutical researchers and clinical practitioners. Key interdisciplinary challenges include establishing standardized *in vitro*-*in vivo* correlation models, defining critical quality attributes for clinical-grade hydrogels and developing noninvasive monitoring techniques for evaluating long-term therapeutic efficacy and safety. A principal direction for future research involves innovating biomaterial design, where developing novel materials with enhanced properties offers considerable potential for advancing hydrogel applications in cartilage repair. Ideal hydrogel scaffolds should demonstrate optimized mechanical performance, excellent biocompatibility and precisely controlled release capabilities for bioactive factors [177, 178]. Advanced fabrication technologies such as nanotechnology and 4D bioprinting enable the engineering of more precise and customizable hydrogel architectures that better recapitulate the native cartilage microenvironment [179]. Biomimetic strategies can further extend hydrogel functionality by incorporating bioinspired signals, including ECM-mimetic biochemical cues and tailored mechanical stimuli, to direct cellular behavior and promote chondrogenic differentiation and tissue maturation. The introduction of topographic features or biochemical gradients within hydrogels may additionally improve the functional integration of engineered cartilage with surrounding native tissue. The integration of stem cell science with tissue engineering represents another critical research frontier. A deeper understanding of hydrogel-stem cell interactions will facilitate the optimization of material properties to enhance cell survival, proliferation and chondrogenic differentiation. Improving the retention, viability and functional activity of transplanted stem cells within hydrogels is expected to significantly improve cartilage regeneration outcomes.

Overall, from the perspective of pharmaceutical science and delivery strategies, this review systematically summarizes the pathological mechanisms of cartilage injury and key design aspects of hydrogels, including the regulation of mechanical properties, drug loading capacity and microenvironment-responsive release behavior. It further discusses rational hydrogel design concepts for treating cartilage defects and reviews recent advances in hydrogels as functional carriers for cartilage regeneration. Finally, the article outlines critical challenges facing next-generation cartilage regeneration strategies and aims to propose a technical pathway for transforming hydrogels into efficient therapies for cartilage repair.
